# Supplementary material for: Prevalence and risk factors of metabolic syndrome in Ethiopia: describing an emerging outbreak in HIV clinics of the sub-Saharan Africa – a cross-sectional study
Source: BMJ Open. 2023 Dec 8;13(12):e069637. doi: 10.1136/bmjopen-2022-069637 (PMC10729149; doi:10.1136/bmjopen-2022-069637)
Supplement: Author's manuscript [file bmjopen-2022-069637.draft_revisions.pdf]

BMJ Open

BMJ Open is committed to open peer review. As part of this commitment we make the peer review history of every article we publish publicly available.

When an article is published we post the peer reviewers' comments and the authors' responses online. We also post the versions of the paper that were used during peer review. These are the versions that the peer review comments apply to.

The versions of the paper that follow are the versions that were submitted during the peer review process. They are not the versions of record or the final published versions. They should not be cited or distributed as the published version of this manuscript.

BMJ Open is an open access journal and the full, final, typeset and author-corrected version of record of the manuscript is available on our site with no access controls, subscription charges or pay-per-view fees (http://bmjopen.bmj.com).

If you have any questions on BMJ Open's open peer review process please email info.bmjopen@bmj.com

BMJ Open

THE PREVALENCE AND RISK FACTORS OF METABOLIC SYNDROME IN ETHIOPIA: DESCRIBING AN EMERGING OUTBREAK IN HIV CLINICS OF THE SUB-SAHARAN AFRICA

Journal:	*BMJ Open*
Manuscript ID	bmjopen-2022-069637
Article Type:	Original research
Date Submitted by the Author:	28-Oct-2022

Complete List of Authors:	Abdela, Abdurezak; Addis Ababa University, Department of Internal Medicine<br>Yifter, Helen; Addis Ababa University, Department of Internal Medicine<br>Reja, Ahmed; Addis Ababa University, Department of Internal Medicine<br>Shewaamare, Aster; Zewditu Memorial Hospital<br>Ofotokun, Ighovwerha; Emory University, Department of Internal Medicine<br>Degu, Wondwossen; Addis Ababa University, Department of Internal Medicine

Keywords:	HIV & AIDS < INFECTIOUS DISEASES, Lipid disorders < DIABETES & ENDOCRINOLOGY, Diabetes & endocrinology < INTERNAL MEDICINE

SCHOLARONE™
Manuscripts

I, the Submitting Author has the right to grant and does grant on behalf of all authors of the Work (as defined in the below author licence), an exclusive licence and/or a non-exclusive licence for contributions from authors who are: i) UK Crown employees; ii) where BMJ has agreed a CC-BY licence shall apply, and/or iii) in accordance with the terms applicable for US Federal Government officers or employees acting as part of their official duties; on a worldwide, perpetual, irrevocable, royalty-free basis to BMJ Publishing Group Ltd ("BMJ") its licensees and where the relevant Journal is co-owned by BMJ to the co-owners of the Journal, to publish the Work in this journal and any other BMJ products and to exploit all rights, as set out in our licence.

The Submitting Author accepts and understands that any supply made under these terms is made by BMJ to the Submitting Author unless you are acting as an employee on behalf of your employer or a postgraduate student of an affiliated institution which is paying any applicable article publishing charge ("APC") for Open Access articles. Where the Submitting Author wishes to make the Work available on an Open Access basis (and intends to pay the relevant APC), the terms of reuse of such Open Access shall be governed by a Creative Commons licence – details of these licences and which Creative Commons licence will apply to this Work are set out in our licence referred to above.

Other than as permitted in any relevant BMJ Author's Self Archiving Policies, I confirm this Work has not been accepted for publication elsewhere, is not being considered for publication elsewhere and does not duplicate material already published. I confirm all authors consent to publication of this Work and authorise the granting of this licence.

For peer review only

THE PREVALENCE AND RISK FACTORS OF METABOLIC SYNDROME IN ETHIOPIA: DESCRIBING AN EMERGING OUTBREAK IN HIV CLINICS OF THE SUB-SAHARAN AFRICA

Word count = 3259

Authors: Abdurezak Ahmed[1], Helen Yifter[1], Ahmed Reja[1], Aster Shewaamare[2], Ighovwerha Ofotokun[3] , Wondwossen Amogne[4]

Corresponding author: Wondwossen Amogne, emial: wonamogne@yahoo.com, Department of Internal Medicine - Addis Ababa University, P.O.Box 2380 Addis Ababa-Ethiopia

[1] Endocrine Unit, Department of Internal Medicine, Addis Ababa University, Ethiopia. Emails in order of authors: abdurezakahmed1977@gmail.com, helenefbr@gmail.com, ahmedreja@yahoo.com
[2] HIV clinic, Zewditu Memorial Hospital, Addis Ababa, Ethiopia. Email: aster.shewaamare@gmail.com
[3]Division of Infectious Diseases, Department of Internal Medicine, Emory University, USA. Email: iofotok@emory.edu
[4] Division of Infectious Diseases, Department of Internal Medicine, Addis Ababa University, Ethiopia. Email:wonamogne@yahoo.com

For peer review only - http://bmjopen.bmj.com/site/about/guidelines.xhtml

ABSTRACT

Objectives

HIV-induced inflammation, immune activation despite achieving undetectable HIV RNA levels, and combined antiretroviral treatment (cART) are associated with metabolic changes that are known to cause cardiovascular diseases. The aims of this study are to estimate the prevalence of metabolic syndrome (MetS), Lipodystrophy, and analyze risk factors in HIV-infected Ethiopian patients receiving cART.

Methods

A multicenter cross-sectional study was conducted at tertiary hospitals. Data were analyzed by SPSS statistical package version 25 with descriptive and analytical statistics. For multivariable analysis of risk factors, logistic regression model was used. Results were presented in frequency and percentages, Mean± Standard Deviation, or Median + interquartile ranges. Statistical significance was taken as $p<0.05$.

Results

Among 518 studied participants, two-third were females and the mean age of the study population was 45 years (±SD=11). The mean duration of cART was 9.97 years(±SD=4). Median recent CD4 count was 460 cells/mm^3. The prevalence of MetS according to the Adult treatment Panel III (2005) criteria was 37.6%. In multivariate analysis, independent risk factors for MetS were age ≥ 45 years (aHR= 1.8, 95% CI 1.2-2.4), female sex (aHR=1.8, 95% CI 1.1-2.8), BMI≥ 25 Kg/m^2 (aHR=2.7, 95% CI 1.8-4.1), Efavirenz-based cART (aHR=2.8, 95% CI 1.6-4.8), and Lopinavir/ritonavir-based cART (aHR=3.7, 95% CI 1.0-13.3). Prevalence of

For peer review only

Lipodystrophy was 23.6%. Prior exposure to Stavudine containing regimen was independently associated with Lipodystrophy (aHR=3.1, 95% CI 1.6-6.1).

Conclusion

About 38 % of the study population had MetS. Independent risk factor for MetS are BMI ≥ 25 kg/m^2, Efavirenz and Lopinavir/ritonavir based cART, female sex, and age ≥ 45 years. In the HIV cohort we examined, the prevalence of MetS is considerably higher. This may indicate enhanced cardiovascular risks. We, therefore, recommend regular screening to identify and treat associated modifiable risk factors to avoid cardiovascular consequences of MetS.

Key words: Metabolic Syndrome, HIV, cART

ARTICLE SUMMARY

Strengths and Limitations of the study

1. This study looks into cardiovascular risk factor, MetS, among HIV patients on cART
2. Of all the studies done in Ethiopia on MetS and Lipodystrophy in HIV patients on cART, this is the one with the largest sample size.
3. Complete nutritional assessment was not made.
4. Advanced techniques for assessment of lipodystrophy were not utilized.

For peer review only

INTRODUCTION

HIV/AIDS is a global pandemic. There was an estimated 36.7 million individuals living with HIV infection by 2015.[1,2] More than 95% of people living with HIV/AIDS reside in low- and middle-income countries.[1]The region of Sub-Saharan Africa where 10–11% of the world's population live is home for more than two-thirds of all people with HIV infection.[1]In the world's most affected region, eastern and southern Africa, the number of people on treatment has more than doubled since 2010, reaching nearly 10.3 million people. AIDS related deaths in the region have decreased by 36% since 2010.[2] This decrease in AIDS-related mortality is mainly attributed to the reduction in opportunistic diseases.

It has been noted that patients with good adherence to combined Antiretroviral therapy (cART) will have a good immunologic response and tend to have a life expectancy similar to non-HIV infected individuals.[3]The Data Collection of Adverse events of Anti-HIV Drugs (D: A: D) study has showed that 289 of 2482 deaths were related to cardiovascular disease (CVD).[4] There is a shift from AIDS related mortality to non-AIDS related mortality as provided by 2006 Antiretroviral therapy cohort collaboration(ART-CC) study showing that the frequency of opportunistic infection as a cause of death in persons living with HIV (PLWH) decreased from 32% in the pre-potent ARV era to 19% thereafter, with the proportion of death increasing from, among others, 1.3% to 4.3% for CVD and diabetes.[5,6]CVD have become of particular concern due to antiviral-drug-induced metabolic changes, the high prevalence of cardiovascular risk factors in HIV-infected individuals, and growing evidence on HIV-accelerated inflammatory processes that are known to promote atherosclerosis.[7]As well, some of antiretroviral drugs are proven to lead to changes in the body appearance with re-distribution of fat. Dyslipidemia is one of the complications of cART. It has a well-established link to an increased risk for

For peer review only

cardiovascular morbidity in PLWH.[8]Metabolic syndrome (MetS), a combination of known cardiovascular risk factors, and HIV associated lipodystrophy have been reported from different countries. [9,10]Metabolic syndrome in HIV patients was detected in 21.1% and HIV associated lipodystrophy was detected in 12.1% of patients. [11] Hypertriglyceredimeia prevalence was 15.2%.[12]Individual components of MetS and metabolic changes with Lipodystrophy are known to be risk factors for CVD.

Since CVD risk factors among HIV patients on cART are of public health importance and the data are scarce about it and fat redistribution, we are reporting on prevalence of MetS, Lipodystrophy, and associated factors among PLWH on cART at two tertiary hospitals, Tikur Anbessa Specialized and Zewditu Memorial Hospitals, from Sub-Saharan Africa HIV clinics in Addis Ababa, Ethiopia.

For peer review only

METHODS

STUDY DESIGN AND ELIGIBILITY

A cross sectional study was conducted at tertiary referral hospitals, Tikur Anbessa Specialized Hospital (TASH) and Zewditu Memorial Hospital (ZMH), in Addis Ababa, Ethiopia. TASH is Addis Ababa University's teaching hospital while ZMH is administered by Addis Ababa Health Bureau. Both hospitals serve large community of PLWH. Addis Ababa has 2.7 million people accounting for nearly 4% of the Ethiopian population.[(13)]TASH and ZMH have a total of 4738 and 7200 HIV patients on cART at the time of the study. Government supported free HIV/AIDS care including cART was delivered in all of these institutions. Institutions based cross sectional study was conducted from July 2018- June 2019.

Study participants were men and women who were HIV/AIDS patients receiving cART from TASH and ZMH, Addis Ababa, Ethiopia. These two hospitals were chosen because they are the largest public HIV care providers in the city for the vast majority of patients.

Patients were excluded if they were less than 18 years of age, withdrawn from cART, diagnosed with Diabetes Mellitus and Hypertension before cART treatment, diagnosed with hypothyroidism, known with Chronic Renal Failure, on corticosteroids, on oral contraception, and were Pregnant. Participants for whom the requirements for the diagnosis of MetS and associated risk factors cannot be obtained because of inability to undergo weight, height, and abdominal circumference measurements, not willing or unable to give a venous blood sample for analysis, and subjects who are too sick to be interviewed and examined were excluded.

Study participants were included if they were above the ages of 18 years, had documented HIV infection and adhering to and taking cART for at least 12 months[(11,12)] before the study period.

For peer review only - http://bmjopen.bmj.com/site/about/guidelines.xhtml

Study subjects were screened by reviewing the subjects' health record and by interview using structured screening questions. A total of 518 subjects were recruited for the study using simple random sampling method.

PATIENT AND PUBLIC INVOLVEMENT

None

DATA COLLECTION AND QUALITY ASSURANCE

Data was collected by trained and qualified HIV specialty clinic nurses by interview on socio demographic characteristics such as age, sex, educational status, income, and occupation. From the patient's health record and data base, data on recent CD4 count and viral load was collected. Data on duration of HIV, duration of treatment with cART, duration of treatment with current cART, type of current cART, history of change of cART, reason for change, type of changed cART, and duration of treatment with changed drug was collected. The rest of clinical questions were also collected by the structured questionnaire. Body fat distribution was assessed by patient's self-report and physical examination. Signs of Lipodystrophy including loss of buccal fat pad, central adiposity with/without extremity wasting, dorsocervical/supraclavicular fat pad, and breast enlargement were documented during physical examination. The height and body weight were measured in centimeters and kilograms, respectively, utilizing a digital balance with height measurement attached to the digital balance. The height measurements were converted into meters. Body mass index (BMI) was calculated as a ratio between the weight in kilograms and the square of the height in meters.(14) Abdominal waist circumference measurement was taken at the approximate midpoint between the lower margin of the last palpable rib and the top of the iliac crest as described in WHO guidelines.(15)

For peer review only

Resting blood pressure in mmHg using an automated sphygmomanometer were measured based on standard definitions.[16]Morning Fasting venous blood sample, 5ml, was collected after an overnight, minimum of eight hours, fasting for fasting blood sugar (FBS), Total Cholestrol (TC), Triglyceride (TG), High Density Lipoprotien-Cholestrol (HDL-C), and Low Density Lipoprotien-Cholestrol (LDL-C) levels determination. Blood samples were collected in Gel and clot activator vacutainer tubes (Henso Medical (Hangzhou) Co.,Ltd.), taken to the lab in the same morning, kept in the room temperature, and analyzed within the same day. Laboratory tests were made for FBS, TC, TG, HDL-C, and LDL-C by direct measurement using reagents(Human Diagnostics worldwide LiquiColor, Germany-Wiesbaden) for all by Mendray chemistry machine. Data was checked for accuracy, consistency, and cleanliness.

ASSESSMENT OF METABOLIC SYNDROME AND LIPODYSTROPHY

MetS was defined using International Diabetes Federation(IDF) [17] and the National Cholesterol Education Program's Adult Treatment Panel (ATPIII)[18] criteria. IDF criteria are waist circumference ≥ 80 cm in women and ≥94 cm in men plus two of the following: triglycerides ≥ 150 mg/dl or drug treatment for high TG , HDL <40 mg/dl in men or <50 mg/dl in women or drug treatment for low HDL-C, FBS ≥ 100 mg/dl or treatment for diagnosed diabetes, or blood pressure ≥130/85 mmHg or drug treatment for hypertension. ATPIII criteria are three of the following: waist circumference ≥88 cm in women and≥102 cm in men, triglycerides ≥ 150 mg/dl or drug treatment for elevated TG, HDL <40 mg/dl in men or <50 mg/dl in women or drug treatment for low HDL-C, FBS ≥ 100 mg/dl or drug treatment for elevated blood glucose, or blood pressure ≥130/85 mmHg or drug treatment for hypertension. Dyslipidemia was defined by ATP III[18] criteria with TC < 200 mg/dl as desirable, TG <150 mg/dl as normal, HDL-C < 40 mg/dl and <50 mg/ dl for men and women respectively as low, and LDL-C <100 mg/dl as

For peer review only

optimal. Lipodystrophy was defined as having any of clinical symptoms or signs, self-reported or on physical examination, of loss of buccal fat pad, abdominal obesity with/without extremity wasting, breast enlargement, or dorsocervical or supraclavicular fat pad accumulation with or without signs of insulin resistance.[19-22]

STATISTICAL ANALYSIS

Data was entered into excel, cleaned, and analyzed by SPSS statistical package version 25. Descriptive statistical analysis was made for sociodemographic and clinical variables and reported as frequency and percent, Mean±SD, or Median + IQR. Prevalence of MetS and Lipodystrophy was computed and reported as percentages. Bivariate analysis was used to identify significant predictors of MetS and Lipodystrophy. Independent variables showing association during bivariate analysis were fed into multivariate models to control for confounding variables and analyzed using multivariate logistic regression. Because only few study participants had the habit of smoking this factor was not tested for association with dependent variables. All significant test values were two sided and significant values considered at $p < 0.05$.

For peer review only

RESULTS

PATIENTS CHARACTERISTICS

A total of 518 men [170 (32.8%)] and women [347 (67.0%)] with Mean± SD age of 45.3±10.74 years were enrolled in this study. Those who have attended primary and secondary school were 120 (23.2%) and 266 (51.4%) respectively. Study participants were mostly private workers and civil servants[(227 (43.8%), 107 (20.7%) respectively]. Largest number of patients were on current regimen of Tenofovir/Lamuvidin/Efavirenz [297 (57.3%)]. Those participants for whom initial cART regimen was changed were 213 (41.1%). Changed initial cART regimens were mostly Stavudine/ Lamuvidine/ Efavirenz and Stavudine/ Lamuvidine/ Nevarapin regimens [69(32.4%)]; and Ziduvodine/ Lamuvidine/ Efavirenz and Ziduvodine/ Lamuvidine/ Nevarapin regimens [91(42.7%)]. The commonest reasons for change of initial cART regimen were treatment failure [68(31.9%)], Lipodystrophy [75(35.2%)],and anemia[26(12.2%)].

Mean± SD duration of HIV, treatment with cART, treatment with current cART, and treatment with initial changed cART in years was 10.8±3.85, 9.97±3.63, 7.90±3.79, and 5.1±3.10 respectively. Mean BMI±SD (kg/m^2)was 24.6±5.02. Mean abdominal waist circumference ±SD (cm) was 90.6±9.48. Median recent CD4 counts (cells/mm^3) was 460 (IQR=326.5,633.5).(Table.1)

For peer review only

Table 1: Sociodemographic and Clinical Characteristics of Study Participants at TASH* and ZMH*, Addis Ababa, Ethiopia

Sociodemographic and Clinical Variables	Frequency	Percent
**Sex**		
Male	170	32.8
Female	347	67.0
**Education**		
No formal Education attended	48	9.30
Primary and Secondary School	386	74.5
Diploma and higher Degree	84	16.2
**Occupation**		
Private works	227	43.8
Civil Servant	107	20.7
Merchant	28	5.40
Housewife	69	13.3
Miscellaneous others	87	16.8
**Types of current cART†**		
[1d,1c,1g,1h,1f]	158	30.5
1e	297	57.3
[2g,2h,2i,2f]	63	12.2
**Initial cART Changed**		
Yes	213	41.1
No	305	58.9
**Types of Changed Initial cART†**		
[1a,1b]	69	32.4
[1c,1d]	91	42.7
[1e,1g,1f]	32	15.0
[2i,2f,2h]	16	7.50
Missing-not traceable	05	2.40
**Reasons for change of cART‡**		
Treatment Failure	68	31.9
Lipodystrophy	75	35.2
Anemia	26	12.2
Others	30	14.1
Missing-not traceable	14	6.60
	**Mean ± SD**	**95% CI for Mean**
**Age**	45.3±10.74	44.37-46.20
**Duration Of:**		
HIV since diagnosed	10.8±3.85	10.46-11.12
Treatment with cART	9.97±3.63	9.65-10.27
Treatment with Current cART	7.90±3.79	7.51-08.19
Treatment with Changed cART	5.10±3.10	4.68-05.58

**BMI (Kg/m²)**	24.6±5.02	23.97-25.40
**Waist Circumference (cm)**	90.6±9.48	89.09-91.90
**Blood Pressure (mmHg)**		
Systolic	122±15.38	119.9-124.3
Diastolic	79.5±10.9	77.96-81.05
	**Median**	**IQR**
**Recent CD4 Count (cells/mm³)**	460	.[326.5, 633.5]

*TASH: Tikur Anbessa Speciaized Hospital, ZMH: Zewditu Memorial Hospital
†cART: combined Antiretroviral Therapy;**1a:** Stavudine (D4T)/ Lamuvidin (3TC) /Nevarapin(NVP); **1b:** D4T/3TC/ Efaverinez (EFV); **1c:** Ziduvidin (ZDV)/3TC/ NVP; **1d:** ZDV/ 3TC/ EFV; **1e**: Tenofivir (TDF)/3TC/EFV; **1f:**TDF/3TC/NVP; **1g:**Abacavir(ABC)/3TC/EFV;**1h:** ABC/3TC/NVP; **2e:**AZT/3TC/ Ritonavir boosted Lopinavir (LPV/r); **2f:**AZT/3TC/ Ritonavir boosted Atazanavir (ATV/r)**; 2g:**TDF/3TC/ (LPV/r); **2h:**TDF/3TC/ ATV/r;**2i:** ABC/3TC/LPV/r
‡ Participants may have more than one reason for change of cART

PREVALENCE OF METABOLIC SYNDROME, LIPODYSTROPHY, AND HYPERTRIGLYCERIDEMIA

Distribution of Lipodystrophy symptoms were loss of buccal fat pad 76(14.7%), central adiposity ±extremity wasting 80(15.4%), dorsocervical/supraclavicular fat pad 39(7.5%), and breast enlargement 31(6.0%). Signs of Lipodystrophy were noted as loss of buccal fat pad 60 (11.6%), central adiposity ±extremity wasting 61(11.8%), dorsocervical/supraclavicular fat pad 1(0.2%).(Table 2)

For peer review only

Table 2: Distribution of Lipodystrophy Symptoms and Signs among Patients on cART* at TASH* and ZMH*, Adddis Ababa, Ethiopia

Variables	Frequency	Percent
**Lipodystrophy Symptoms**		
**Loss of buccal fat Pad**		
Yes	76	14.7
No	442	85.3
**Central adiposity ±extremity wasting**		
Yes	80	15.4
No	438	84.6
**Supraclavicular/dorsocervical fat pad**		
Yes	39	7.5
No	479	92.5
**Breast Enlargement**		
Yes	31	6.0
No	486	93.8
**Lipodystrophy Signs**		
Loss of buccal fat pad	60	11.6
Central adiposity ±extremity wasting	61	11.8
Supraclavicular/dorsocervical fat pad	1	0.2
No Sign of Lipodystrophy	396	76.4

*cART: combined Antiretroviral Therapy, TASH: Tikur Anbessa Specialized Hospital, ZMH: Zewditu Memorial Hospital

In this cohort of 518 study participants, prevalence of MetS by IDF 2006 criteria was found to be 193 (37.3%) and by ATPIII 2005 criteria 195(37.6%). Prevalence of Lipodystrophy was 22.6% (117 cases) by symptoms and 23.6% (122 cases) by signs. Prevalence of Hypertriglecridemia was 40.7% (211 cases).(Table 3).

For peer review only

Table 3: Prevalence of Metabolic Syndrome, Lipodystrophy, and hypertriglyceridemia among HIV patients on cART* at TASH* and ZMH*, Addis Ababa, Ethiopia

Variables	Frequency	Percent
**Metabolic Syndrome IDF 2006**		
**Yes**	**193**	**37.3**
**No**	**325**	**62.7**
**Metabolic Syndrome ATP III 2005**		
**Yes**	**195**	**37.6**
**No**	**323**	**62.4**
**Lipodystrophy**		
**Symptoms**		
**Yes**	**117**	**22.6**
**No**	**401**	**77.4**
**Signs**		
**Yes**	**122**	**23.6**
**No**	**396**	**76.4**
**Hypertrigleceridemia**		
**Yes**	**211**	**40.7**
**No**	**307**	**59.3**

*cART: combined Antiretroviral Treatment, TASH: Tikur Anbessa Specialized Hospital, ZMH: Zewditu Memorial Hospital

ASSOCIATED RISK FACTORS FOR METABOLIC SYNDROME AND LIPODYSTROPHY

Bivariate regression analysis has revealed significant association of age ≥45 years [cHR=1.68, 95%CI = 1.1-2.5, p=0.009] compared to age < 45 years, female sex [cHR=1.71, 95%CI=1.2-2.5, p=0.003] compared to male sex, BMI(kg/m^2)≥ 25 [cHR=3.1, 95%CI = 2.1-4.4), p=0.001] compared to BMI ≤ 24.9, and EFV based cART regimen [cHR = 2.8, 95%CI= 1.71-4.65, p=0.001] compared to NVP based regimen with MetS based on IDF 2006 criteria. In comparison to Nevirapin (NVP) based-regimen, Lopinavir-based regimen had cHR = 2.8, 95% CI = 0.94-8.26. Independently associated risk factors for MetS on multivariate regression

For peer review only

analysis were age ≥45 years [aHR=1.8, 95%CI= 1.2-2.4, p=0.006] versus age <45years, female sex[aHR= 1.8, 95%CI= 1.1-2.8, p=0.013] versus male sex, BMI (kg/m^2) ≥ 25 [aHR= 2.71 , 95%CI= (1.8-4.1), p=0.001] versus BMI≤ 24.9 kg/m^2, EFV based cART regimen [aHR=2.8 95%CI = 1.6-4.8, p=0.001] compared to NVP based regimen, and LPV based cART regimen [aHR= 3.7, 95%CI = 1.0-13.3), p=0.043] compared to NVP based regimen. CD4 count above 350 cells/mm^3 was not significantly associated with MetS in this study [cHR= 1.7, 95%CI = 0.8-3.6), p=0.143, aHR =1.5, 95%CI= 0.7-3.4%), p=0.320].(Table.4)

Table 4: Associated Risk Factors for Metabolic Syndrome among HIV patients on cART* at TASH* and ZMH*, Addis Ababa, Ethiopia

	Metabolic Syndrome †		cHR (95%CI)	P-Value	aHR (95%CI)	P-Value
**Variables**	Yes	No				
**Age (yrs)**						
**<45**	80(31.0%)	178(69.0%)	1		1	
**≥45**	113(43.5)	147(56.5%)	1.68 (1.14-2.49)	0.009	1.79(1.18 -2.37)	0.006
**Sex**						
**Male**	50 (29.4%)	120 (70.6%)	1		1	
**Female**	143(41.2%)	204 (58.8%)	1.71(1.19 -2.45)	0.003	1.79(1.13 - 2.83)	0.013
**Recent CD4 Count (cells/mm^3)**						
**<200**	11 (29.0%)	27 (71.0%)	1		1	
**200-350**	30 (29.4%)	72 (70.6%)	1.02 (0.45-2.32)	0.957	0.99(0.41-2.40)	0.989
**>350**	143(41.3%)	203(59.7%)	1.73(0.83-3.60)	0.143	1.50(0.67-3.35)	0.320
**BMI (kg/m^2)**						
**≤24.9**	83 (27.0%)	225(73.0%9	1		1	
**≥25**	108 (53.0%)	6 (47.0%)	3.05(2.10-4.43)	0.001	2.71(1.81-4.06)	0.001
**Current cART‡**						
**NVP based**	24(21.8%)	86(79.2%)	1		1	
**EFV based**	152(44.1%)	193(55.9%)	2.82(1.71-4.65)	0.001	2.79(1.62-4.80)	0.001
**LPV based**	7 (43.7%)	9 (56.3%)	2.79(0.94-8.26)	0.640	3.72(1.04-13.3)	0.043
**ATV based**	10 (21.3%)	37(78.7%)	0.97(0.42-2.23)	0.940	1.03(0.41-2.60)	0.954

* cART: combined Antiretroviral Treatment, TASH: Tikur Anbessa Specialized Hospital, ZMH: Zewditu Memorial Hospital

For peer review only

†Based on IDF 2006 Metabolic Syndrome Diagnostic Criteria

‡Current cART: NVP: Nevirapin, EFV:Efavirenz, LPV: Lopinavir, ATV: Atazanavir

Associated risk factors for Lipodystrophy on bivariate analysis were Stavudine containing regimen exposure [cHR= 3.1, 95%CI= 1.7-5.7, p=0.001] compared to non exposure to Stavudine, BMI(kg/m^2) ≥ 25 [cHR= 2.8, 95%CI= 1.8-4.2, p=0.001] compared to BMI (kg/m^2) ≤ 24.9. Independently associated risk factor for Lipodytrophy was Stavudine based cART regimen in this study [aHR= 3.1, 95%CI= 1.6-6.1, p=0.001]. There was no significant association between CD4 count and recent cART regimen and Lipodystrophy in this study.(Table 5)

Table 5: Associated Risk factors for Lipodystrophy among HIV Patients on cART* at TASH*and ZMH*, Addis Ababa, Ethiopia

	Lipodystrophy Sign(s)		cHR (95%CI)	*p*-value	aHR (95%CI)	*p*-Value
**Variables**	**Yes**	**No**				
**Initial cART†**						
**D4T Exposed**	36(52.2%)	33(47.8%)	3.09(1.68-5.68)	0.001	3.13(1.6-6.12)	0.001
**D4T Not exposed**	35(26.1%)	99(73.9%)	1		1	
**CD4 Count (cells/mm^3)**						
**<200**	6(16.7%)	30(83.3%)	1		1	
**200-350**	18(17.3%)	86(82.7%)	1.14(0.42-3.14)	0.795	0.63(0.18-2.18)	0.462
**>350**	92(26.6%)	254(73.4%)	1.93(0.78-4.77)	0.153	0.62(0.27-1.41)	0.254
**BMI* (kg/m^2)**						
**≤24.9**	71(34.7%)	133(65.2%)	1		1	
**≥25**	50(16.2%)	258(83.8%)	2.76(1.81-4.18)	0.001	1.48(0.76-2.86)	0.249
**Current cART‡**						
**NVP based**	24(21.8%)	86(78.2%)	1		1	
**EFV based**	85(24.6%)	260(75.4%)	1.17(0.70-1.96)	0.547	1.20(0.52-2.78)	0.666
**LPV based**	0	16(100%)	0.00(00-)	0.998	0.00(0.00-)	0.999
**ATV based**	13(27.7%)	34(72.3%)	1.37(0.63-3.00)	0.431	0.90(0.29-2.77)	0.857

*TASH: Tikur Anbessa Specialized Hospital, ZMH: Zewditu Memorial Hospital, BMI: Body Mass Index, cART: combined Antiretroviral Therapy
†Intial cART regimen: for those participants whose drugs were changed to the current cART regimen ; D4T : Stavudine
‡Current cART: NVP: Nevirapin, EFV:Efavirenz, LPV: Lopinavir, ATV: Atazanavir

For peer review only

DISCUSSION

The current study has found prevalence of MetS to be 37.3% by IDF 2006 criteria [17] and 37.6% by ATP III 2005 criteria.[18] In comparison to the hypertensive none HIV population in Gonder-Ethiopia by Tachebele etal [23] [40.7% IDF and 39.3% ATPIII], our finding was slightly lower. The prevalence of MetS was higher by both criteria in our study than the finding by Samaras and colleagues,[24] [14% (*n* _ 114; 83 men)] by IDF criteria and [18% (*n*_139; 118 men)] by ATPIII criteria, and Berhane and colleagues[11] from Southern Ethiopia [21.1%] by IDF criteria. This study finding for prevalence of MetS is also higher than the Tanzanian study [25] that revealed a 25.6% prevalence using IDF criteria.

This study finding of prevalence of MetS is lower than the Italian study that reported 45.4% prevalence by ATPIII criteria.[26]Compared to the most recent data[27] with prevalence of MetS using IDF definition of 4.8% (8.6% in females and vs. 1.8% in males) in national NCD STEPS survey in Ethiopia in general population, our study finding may indicate significantly higher risk of CVD among this group of patients. The disparity in prevalences might be attributed to some extent to the duration between the studies, the types of cART in use during the studies, and the populations studied. Even though the IDF criteria appears to be more stringent because of the requirement of waist circumference, it also allows more patients with lesser waist circumference to be included as it became evident in this result showing a closer prevalence to the ATP III criteria. Similar to our results, Samaras and colleagues [24] have found a higher prevalence of MetS by ATPIII criteria. Unlike our result, slightly higher IDF criteria finding of MetS was found by Hirigo and Tesfaye[28] from Southern Ethiopia with overall prevalence of MetS 24.3% and 17.8 % using IDF and NCEP-ATP III criteria respectively.

For peer review only

Independently associated risk factors for MetS in our study after adjustment for CD4 count were age ≥45 years versus age <45years, female sex versus male sex, BMI (kg/m^2) ≥ 25 versus BMI≤ 24.9 kg/m^2, EFV based cART regimen compared to NVP based regimen, and LPV based cART regimen compared to NVP based regimen. Similar to our finding, studies in Ethiopia[(28)] and Tanzania[(25)] have found a significant association between female sex and MetS. This is in line with other reports.[(24,29)] In contrast, one study revealed that gender has no significant association with MetS.[(30)]. Similar to our study findings, Hirigo and Tesfay[(28)]have reported an increasing age above 40 years to be significantly associated with MetS. This is in line with the report from Spain that has showed association between increasing age and MetS.[(24)] Similar to our study finding, higher BMI was significantly associated with MetS in other studies.[(24,28)]Furthermore, several other studies illustrated that the BMI is a quantitative predictor of MetS.[(31-33)] Current use of EFV based regimen has been found to be significantly associated with MetS in our study. This is supported by the fact that nonucleoside reverse transcriptase inhibitors impair lipid metabolism as a class[(34)]and Efavirenz leads to increased inflammation,[(35,36)] impaired adipogenesis and adipocyte differentiation,[(35)] impaired metabolism, [(35,36)] and no mitochondrial toxicity.[(36)]Current EFV treatment, has been correlated with elevated markers of atherosclerosis.[(37)]The finding of Liponavir based cART regimen is in line with the independent association with MetS in study in Barcelona, Spain.[(24)]This is supported by the molecular mechanism of the drug that causes substantial impairment of adipocyte differentiation and lipid or glucose metabolism, pronounced decrease in mitochondrial proteins, and increased inflammation.[(38)]

Prevalence of Lipodystrophy in this study was found to be 22.6% by subjective participants report and 23.6% by objective assessment for signs. Our study finding is higher than the report of

12.1% by Berhane etal[11] from South West Ethiopia and lower than 68.3% prevalence in the study[12] from Addis Ababa. In our study, the higher prevalence compared to Berhane etal's[11] finding might be attributable to the larger sample size and the lower prevalence compared to Feleke etal's[12] finding may be explained by the trends of cART drug prescription that has been towards withdrawal of Stavudine. In our study the prevalence of hypertriglyceridemia ≥ 150 mg/dl, a marker of Lipodystrophy with insulin resistance and a component of MetS, was found in 40.7% of participants. This finding is closer to the finding of 43.5% prevalence in urban Tanzanian HIV population on cART[25], higher than 15.2%[12] prevalence finding in Addis Ababa, and lower than the report of 59.6% prevalence in a study in defense hospital in Addis Ababa.[39] Unadjusted, Lipodystrophy was significantly associated with Stavudine containing regimen and BMI (kg/m^2) ≥ 25. However, after adjustment for CD4 count (cells/mm^3) and current cART, only D4T exposure was significantly associated with Lipodystrophy. This finding of strong association between D4T and MetS is in line with previous reports.[24,28] This has been shown to be because of nucleoside reverse transcriptase class effect on impairing lipid metabolism[34] and D4T effect of reduction of mitochondrial DNA.[40,41]

Limitations of this study are the cross sectional design, difficulty to trace some of the records on the initial cART regimen from old records, difficulty to get adequate samples for some of the variables that may have influenced the outcome like patients on Atazanavir based regimen, and fewer patients who were smokers or taking alcohol to identify their potential correlation in the study participants, and slightly fewer patients on Liponavir based regimen as well. Besides, complete nutritional assessment was not made. Advanced techniques for assessment of lipodystrophy were not utilized because of availability. Of all the studies done in Ethiopia on

For peer review only

MetS and Lipodystrophy, this is the one with the largest sample size and laboratory test results availability for all participants.

CONCLUSION

MetS was detected in 37.3% and 37.6% in IDF and ATPIII criteria respectively. According to IDF criteria, the prevalence of MetS was significantly higher among females compared to males, and among those above 45 years old. As well, higher BMI, EFV based currently used cART regimen, and current use of Liponavir based cART regimens were significantly associated with MetS. As well, hypertriglyceridemia was highly prevalent among patients on cART indicating significant lipid abnormality and insulin resistance. Lipodystrophy was detected in 23.6% of study participants. Previous Stavudine exposure was significantly associated with lipodystrophy. Metabolic changes among patients on cART may put them at high risk of cardiovascular diseases. Therefore, screening for MetS in HIV patients on cART and management of modifiable CV risk factors are recommended. Furthermore, a prospective cohort study to evaluate MetS and Lipodystrophy and the CV outcomes related to them among patients on cART are recommended.

AUTHOR CONTRIBUTION

AA, IO, WA conceptualized the study. AA collected the primary data, conducted the analysis, and drafted the manuscript. HY, AR, AS, IO, WA contributed to conceptualization, collection of data, and data analysis. AA, HY, AR, AS, IO, WA have read, reviewed, and approved the article.

FUNDING

This collaborative research work is financially supported by an NIH funded project, Medical Education partnership Initiative- Junior Faculty (D43TW010143).

For peer review only

ACKNOWLEDGMENT

The authors acknowledge study participants for their voluntary participation in the study. We are also grateful for data collectors and the study centers, ZMH and TASH, for their support in conduct of the study. In addition, the authors acknowledge Dorqa Woldesenbet (MPH) from school of public health-Addis Ababa University for her support in statistical analysis.

CONFLICT OF INTEREST

The authors declare that this research was conducted in the absence of any commercial or financial conflict of interest.

ETHICAL CONSIDERATIONS

The study has been reviewed and approved by the Institution Review Board of the College of Health Sciences- Addis Ababa University with the Ref/ID# 077/17/IM and have therefore been performed in accordance with the ethical standards of Declaration of Helsinki.

For peer review only

REFERENCES:

1. Kasper LD, Fauci SA, Hauser SL, Longo DL, Jameson LJ, Loscalzo; Harrison's Principles of Internal Medicine- 18th edition. 2012. New York. McGraw-Hill Medical Publishing Division.
2. Global AIDS UPDATES; UNAIDS 2016
3. Obel N, Omland LH, Kronborg G *et al.* Impact of non-HIV and HIV risk factors on survival in HIV-infected patients on HAART: a population-based nationwide cohort study. *PLoS ONE*6, E22698 (2011).
4. Data Collection on Adverse Events of Anti H. I. V. drugs Study Group, Smith C, Sabin CA, Lundgren JD, etal. Factors associated with specific causes of death amongst HIV-positive individuals in the D:A:D study. AIDS 2010; 24: 1537–1548.
5. Smit C, Geskus R, walker S, et al. effective therapy has altered the spectrum of cause specific mortality following HIV seroconversion. AIDS. 2006;20:741-749
6. Samji H, Cescon A, Hogg RS, et al. Closing the Gap: increase in life expectancy among treated HIV positive individuals in the United States and Canada. PLoSone. 2013; 8(12):e81355
7. Lars G. Hemkens ,Heiner C. Bucher; novel therapeutic concepts, HIV infection and cardiovascular disease; European Heart journal , REVIEW; advance access published January 9,2014; doi:10.1093/euroheartj/eht528
8. Grover SA, Coupal L, Gilmore N, Mukherjee J. Impact of dyslipidemia associated with highly active antiretroviral therapy oncardiovascular risk and life expectancy. Am J Cardiol. 2005 Mar 1;95(5):586-91. This article on PubMed

For peer review only

9. Domingo P, Sambeat MA, Pérez A, Ordoñez J, Rodriguez J, Vázquez G. Fat distribution and metabolic abnormalities in HIV-infectedpatients on first combination antiretroviral therapy including stavudine or zidovudine: role of physical activity as a protective factor.AntivirTher. 2003; 8(3):223-231. This article on PubMed

10. Calza L, Manfredi R, Chiodo F. Dyslipidaemia associated with antiretroviral therapy in HIV-infected patients. J Antimicrob Chemother.2004;53(1):10-14. This article on PubMed

11. Tsegaye Berhane etal; prevalence of lipodystrophy and Metabolic syndrome among HIV positive individuals on HAART in Jimma, south west Ethiopia; Pan African Medical Journal. 2012; 13:43

12. Feleke Y, Fekade D, Mezgebu Y; prevalence of HAART associated metabolic abnormalities and lipodystrophy in HIV infected patients;Ethiopian medical Journal:2012, 50(3):221-230

13. Federal Democratic republic of Ethiopia, Ministry of Health: health sector development program IV 2010/11-2014/15.2010

14. Gibson RS. Principles of nutritional assessment. New York, Oxford University Press; 2005.15.

15. WHO. WHO STEPwise approach to surveillance (STEPS). Geneva: World Health Organization (WHO); 2008.

16. Perloff D, Grim C, Flack J et al. Human blood pressure determination by sphygmomanometry. Circulation. 1993;88:2460-2470.

17. International Diabetes Federation. The IDF consensus worldwide definition of the metabolic syndrome, 2006. file://www.idf.org/webdata/docs/MetS_def_update2006.pdf

For peer review only

18. Grundy SM, Brewer HB Jr, Cleeman JI, Smith SC Jr, Lenfant C; American Heart Association; National Heart, Lung, and Blood Institute. Definition of metabolic syndrome: Report of the National Heart, Lung, and Blood Institute/American Heart Association conference on scientific issues related to definition. Circulation. 2004; 109:433- 438. This article on PubMed

19. Freitas P, Santos AC, Carvalho Det al. Fat mass ratio: an objective tool to define lipodystrophy in HIV-infected patients under antiretroviral therapy. J Clin Densitom. 2010;13:197–203.

20. Bacchetti P, Gripshover B, Grunfeld C, et al. Fat distribution in men with HIV infection. J Acquir Immune Defic Syndr. 2005;40:121–31.

21. Jantarapakde J, Phanuphak N, Chaturawit C, et al. Prevalence of metabolic syndrome among antiretroviral-naive and antiretroviral-experienced HIV-1 infected Thaiadults. AIDS Patient Care STDS. 2014;28:331–40.

22. Carr A, Samaras K, Thorisdottir A, Kaufmann GR, Chisholm DJ, Cooper DA. Diagnosis, prediction, and natural course of HIV-1 protease-inhibitor associated lipodystrophy, hyperlipidaemia, and diabetes mellitus: a cohort study. Lancet. 1999;353:2093–9.

23. Belaynesh Tachebele, Molla Abebe, Zelalem Addis, and Nebiyu Mesfin; Metabolic syndrome among hypertensive patients at University of Gondar Hospital, North West Ethiopia: a cross sectional study; BMC Cardiovascular Disorders 2014, 14:177

24. Samaras K, Wand H, Law M, Emery S, Cooper D, Carr A. Prevalence of metabolic syndrome in HIV-infected patients receiving highly active antiretroviral therapy using international diabetes foundation and adult treatment panel III criteria. Diabetes Care. 2007;30:113–9.

For peer review only

25. Kagaruki G, Kimaro G, Mweya C etal.; Prevalence and Risk Factors of Metabolic Syndrome among Individuals Living with HIV and Receiving Antiretroviral Treatment in Tanzania; British Journal of Medicine & Medical Research 5(10): 1317-1327, 2015, Article no.BJMMR.2015.150 ISSN: 2231-0614

26. Gazzaruso C, Sacchi P, Garzaniti A, Fratino P, Bruno R, Filice G: Prevalence of metabolic syndrome among HIV patients. Diabetes Care 2002, 25:1253–1254.

27. Gebreyes Y, Goshu D, Geleta T etal; Prevalence of high bloodpressure, hyperglycemia, dyslipidemia, metabolic syndrome and their determinants in Ethiopia: Evidences from the National NCDs STEPS Survey, 2015 PLOS ONE | https://doi.org/10.1371/journal.pone.0194819 May 9, 2018

28. Agete Tadewos Hirigo, Demo Yemane Tesfaye; Influences of gender in metabolic syndrome and its components among people living with HIV virus using antiretroviral treatment in Hawassa, southern Ethiopia; *BMC Res Notes (2016) 9:145* DOI 10.1186/s13104-016-1953-2

29. Ivana K, Ariana V, Branka S, Mladen S, Josipa K, Silvije V. Metabolic syndrome in a metapopulation of Croatian island isolates. Croat Med J. 2006;47:585–92.

30. Lauda LG, Mariath AB, Grillo LP. Metabolic syndrome and its components in HIV-infected individuals. Rev Assoc Med Bras. 2011;57:178–81.

31. Jacobson DL, Tang AM, Spiegelman D, etal. Incidence of metabolic syndrome in a cohort of HIVinfected adults and prevalence relative to the US population (National Health and Nutrition Examination Survey). J Acquir Immune Defic Syndr. 2006;43:458–66.

32. Jericó C, Knobel H, Montero M, et al. Metabolic syndrome among HIV-infected patients: prevalence, characteristics, and related factors. Diabetes Care. 2005;28(132):137.

33. Mondy K, Overton ET, Grubb J etal.. Metabolic syndrome in HIV-infected patients from an urban, midwestern US outpatient population. Clin Infect Dis. 2007;44:726–73.

34. Minami R, Yamamoto M, Takahama S, Ando H, Miyamura T, Suematsu E. Comparison of the infl uence of four classes of HIV antiretrovirals on adipogenic diff erentiation: the minimal eff ect of raltegravir and atazanavir. *J Infect Chemother* 2011; 17: 183–88.

35. Diaz-Delfi n J, Domingo P, Mateo MG, et al. Effects of rilpivirine on human adipocyte diff erentiation, gene expression, and release of adipokines and cytokines. *Antimicrob Agents Chemother* 2012;56: 3369–75.

36. Diaz-Delfi n J, del Mar Gutierrez M, Gallego-Escuredo JM, et al. Eff ects of nevirapine and efavirenz on human adipocyte diff erentiation, gene expression, and release of adipokines andcytokines. *Antiviral Res* 2011; 91: 112–19.

37. Rudolph L. Gleason Jr., Alexander W. Cualk, Daniel Seifu, etal; Current Efavirenz (EFV) or Ritonavir-Boosted Lopinavir (LPV/r) Use Correlates with Elevate Markers of Atherosclerosis in HIV-Infected Subjects in Addis Ababa, Ethiopia; PLOS ONE | DOI:10.1371/journal.pone.0117125 April 27, 2015

38. Capel E, Auclair M, Caron-Debarle M, Capeau J. Effects of ritonavirboosteddarunavir, atazanavir and lopinavir on adipose functionsand insulin sensitivity in murine and human adipocytes.*Antivir Ther* 2012; 17: 549–56.

For peer review only

39. Bayenes H, Ahmed M, Shenkute T, etal.;Prevalence and predictors of dyslipidemia on HAART and HAART naive HIV positive persons in Defense Hospital, Addis Ababa, Ethiopia; American Journal of Health Research.2014; 2(5): 303-309

40. Feeney ER, van Vonderen MG, Wit F, et al. Zidovudine/lamivudine but not nevirapine in combination with lopinavir/ritonavir decreases subcutaneous adipose tissue mitochondrial DNA. *AIDS* 2012; 26: 2165–74.

41. McComsey GA, Daar ES, O'Riordan M, et al. Changes in fat mitochondrial DNA and function in subjects randomized to abacavir-lamivudine or tenofovir DF-emtricitabine with atazanavirritonavir or efavirenz: AIDS Clinical Trials Group study A5224s, substudy of A5202. *J Infect Dis* 2013; 207: 604–11.

For peer review only

Reporting checklist for cross sectional study.

Based on the STROBE cross sectional guidelines.

Instructions to authors

Complete this checklist by entering the page numbers from your manuscript where readers will find each of the items listed below.

Your article may not currently address all the items on the checklist. Please modify your text to include the missing information. If you are certain that an item does not apply, please write "n/a" and provide a short explanation.

Upload your completed checklist as an extra file when you submit to a journal.

In your methods section, say that you used the STROBE cross sectionalreporting guidelines, and cite them as:

von Elm E, Altman DG, Egger M, Pocock SJ, Gotzsche PC, Vandenbroucke JP. The Strengthening the Reporting of Observational Studies in Epidemiology (STROBE) Statement: guidelines for reporting observational studies.

		Reporting Item	Page Number
**Title and abstract**			
Title	#1a	Indicate the study's design with a commonly used term in the title or the abstract	2
Abstract	#1b	Provide in the abstract an informative and balanced summary of what was done and what was found	2
**Introduction**			
Background / rationale	#2	Explain the scientific background and rationale for the investigation being reported	4
Objectives	#3	State specific objectives, including any prespecified hypotheses	5
**Methods**			
Study design	#4	Present key elements of study design early in the paper	6
Setting	#5	Describe the setting, locations, and relevant dates, including periods of recruitment, exposure, follow-up, and data collection	6
Eligibility criteria	#6a	Give the eligibility criteria, and the sources and methods of selection of participants.	6

For peer review only

	#7	Clearly define all outcomes, exposures, predictors, potential confounders, and effect modifiers. Give diagnostic criteria, if applicable	8
Data sources / measurement	#8	For each variable of interest give sources of data and details of methods of assessment (measurement). Describe comparability of assessment methods if there is more than one group. Give information separately for for exposed and unexposed groups if applicable.	6,7,8
Bias	#9	Describe any efforts to address potential sources of bias	7
Study size	#10	Explain how the study size was arrived at	7
Quantitative variables	#11	Explain how quantitative variables were handled in the analyses. If applicable, describe which groupings were chosen, and why	9
Statistical methods	#12a	Describe all statistical methods, including those used to control for confounding	9
Statistical methods	#12b	Describe any methods used to examine subgroups and interactions	9
Statistical methods	#12c	Explain how missing data were addressed	n/a
Statistical methods	#12d	If applicable, describe analytical methods taking account of sampling strategy	n/a
Statistical methods	#12e	Describe any sensitivity analyses	n/a
Results			
Participants	#13a	Report numbers of individuals at each stage of study—eg numbers potentially eligible, examined for eligibility, confirmed eligible, included in the study, completing follow-up, and analysed. Give information separately for for exposed and unexposed groups if applicable.	10
Participants	#13b	Give reasons for non-participation at each stage	n/a
Participants	#13c	Consider use of a flow diagram	n/a
Descriptive data	#14a	Give characteristics of study participants (eg demographic, clinical, social) and information on exposures and potential confounders. Give information separately for exposed and unexposed groups if applicable.	10, 11
Descriptive data	#14b	Indicate number of participants with missing data for each variable of interest	11
Outcome data	#15	Report numbers of outcome events or summary measures. Give information separately for exposed and	12,13,14

For peer review only

		unexposed groups if applicable.	
Main results	#16a	Give unadjusted estimates and, if applicable, confounder-adjusted estimates and their precision (eg, 95% confidence interval). Make clear which confounders were adjusted for and why they were included	14,15,16
Main results	#16b	Report category boundaries when continuous variables were categorized	n/a
Main results	#16c	If relevant, consider translating estimates of relative risk into absolute risk for a meaningful time period	n/a
Other analyses	#17	Report other analyses done—e.g., analyses of subgroups and interactions, and sensitivity analyses	n/a
Discussion			
Key results	#18	Summarise key results with reference to study objectives	17,18,19
Limitations	#19	Discuss limitations of the study, taking into account sources of potential bias or imprecision. Discuss both direction and magnitude of any potential bias.	19
Interpretation	#20	Give a cautious overall interpretation considering objectives, limitations, multiplicity of analyses, results from similar studies, and other relevant evidence.	20
Generalisability	#21	Discuss the generalisability (external validity) of the study results	20
Other Information			
Funding	#22	Give the source of funding and the role of the funders for the present study and, if applicable, for the original study on which the present article is based	20

None The STROBE checklist is distributed under the terms of the Creative Commons Attribution License CC-BY. This checklist can be completed online using https://www.goodreports.org/, a tool made by the EQUATOR Network in collaboration with Penelope.ai

For peer review only

BMJ Open

THE PREVALENCE AND RISK FACTORS OF METABOLIC SYNDROME IN ETHIOPIA: DESCRIBING AN EMERGING OUTBREAK IN HIV CLINICS OF THE SUB-SAHARAN AFRICA. A CROSS SECTIONAL STUDY

Journal:	*BMJ Open*
Manuscript ID	bmjopen-2022-069637.R1
Article Type:	Original research
Date Submitted by the Author:	04-Aug-2023
Complete List of Authors:	Abdela, Abdurezak; Addis Ababa University, Department of Internal Medicine<br>Yifter, Helen; Addis Ababa University, Department of Internal Medicine<br>Reja, Ahmed; Addis Ababa University, Department of Internal Medicine<br>Shewaamare, Aster; Zewditu Memorial Hospital<br>Ofotokun, Ighovwerha; Emory University, Department of Internal Medicine<br>Degu, Wondwossen; Addis Ababa University, Department of Internal Medicine
**Primary Subject Heading**:	Diabetes and endocrinology
Secondary Subject Heading:	HIV/AIDS
Keywords:	HIV & AIDS < INFECTIOUS DISEASES, Lipid disorders < DIABETES & ENDOCRINOLOGY, Diabetes & endocrinology < INTERNAL MEDICINE

SCHOLARONE™
Manuscripts

I, the Submitting Author has the right to grant and does grant on behalf of all authors of the Work (as defined in the below author licence), an exclusive licence and/or a non-exclusive licence for contributions from authors who are: i) UK Crown employees; ii) where BMJ has agreed a CC-BY licence shall apply, and/or iii) in accordance with the terms applicable for US Federal Government officers or employees acting as part of their official duties; on a worldwide, perpetual, irrevocable, royalty-free basis to BMJ Publishing Group Ltd ("BMJ") its licensees and where the relevant Journal is co-owned by BMJ to the co-owners of the Journal, to publish the Work in this journal and any other BMJ products and to exploit all rights, as set out in our licence.

The Submitting Author accepts and understands that any supply made under these terms is made by BMJ to the Submitting Author unless you are acting as an employee on behalf of your employer or a postgraduate student of an affiliated institution which is paying any applicable article publishing charge ("APC") for Open Access articles. Where the Submitting Author wishes to make the Work available on an Open Access basis (and intends to pay the relevant APC), the terms of reuse of such Open Access shall be governed by a Creative Commons licence – details of these licences and which Creative Commons licence will apply to this Work are set out in our licence referred to above.

Other than as permitted in any relevant BMJ Author's Self Archiving Policies, I confirm this Work has not been accepted for publication elsewhere, is not being considered for publication elsewhere and does not duplicate material already published. I confirm all authors consent to publication of this Work and authorise the granting of this licence.

For peer review only

THE PREVALENCE AND RISK FACTORS OF METABOLIC SYNDROME IN ETHIOPIA: DESCRIBING AN EMERGING OUTBREAK IN HIV CLINICS OF THE SUB-SAHARAN AFRICA. A CROSS SECTIONAL STUDY

For peer review only

Word count = 3979

Authors: Abdurezak Ahmed[1], Helen Yifter[1], Ahmed Reja[1], Aster Shewaamare[2], Ighovwerha Ofotokun[3] , Wondwossen Amogne[4]

Corresponding author: Wondwossen Amogne, emial: wonamogne@yahoo.com, wondwossen.amogne @aau.edu.et, Department of Internal Medicine, Addis Ababa University, P.O.Box 2380 Addis Ababa-Ethiopia

[1] Endocrine Unit, Department of Internal Medicine, Addis Ababa University, Ethiopia. Emails in order of authors: abdurezakahmed1977@gmail.com, helenefbr@gmail.com, ahmedreja@yahoo.com
[2] HIV clinic, Zewditu Memorial Hospital, Addis Ababa, Ethiopia. Email: aster.shewaamare@gmail.com
[3]Division of Infectious Diseases, Department of Internal Medicine, Emory University, USA. Email: iofotok@emory.edu
[4] Division of Infectious Diseases, Department of Internal Medicine, Addis Ababa University, Ethiopia. Email:wonamogne@yahoo.com, wondwossen@aau.edu.et

ABSTRACT

Objectives

HIV-induced chronic inflammation, immune activation, and combination antiretroviral therapy (cART) are linked with adverse metabolic changes known to cause cardiovascular adversities. This study evaluates the prevalence of lipodystrophy, and metabolic syndrome (MetS), and analyzes risk factors in HIV-infected Ethiopians taking cART.

Methods

A multicenter cross-sectional study was conducted at tertiary-level hospitals. Eligible participants attending the HIV clinics were enrolled. Sociodemographic, anthropometric, clinical, HIV treatment variables, lipid profile, fasting blood glucose level, risk factors, and components of MetS, also lipodystrophy were studied. Data were analyzed by SPSS statistical package version 25 with descriptive and analytical statistics. For multivariable analysis of risk factors, a logistic regression model was used. Results were presented in frequency and percentages, Mean± Standard Deviation, or Median + interquartile ranges. Statistical significance was taken as $p<0.05$.

Results

Among 518 studied participants, two-thirds were females, and the mean age of the study population was 45 years (SD=11). The mean duration of cART was 10 years(SD=4). Median CD4 count was 460 cells/mm^3. The prevalence of MetS according to the Adult Treatment Panel III (2005) criteria was 37.6%. In multivariable analysis, independent risk factors for MetS were age ≥ 45 years (aHR= 1.8, 95% CI 1.2-2.4), female sex (aHR=1.8, 95% CI 1.1-2.8), BMI≥ 25 Kg/m^2 (aHR=2.7, 95% CI 1.8-4.1), efavirenz-based cART (aHR=2.8, 95% CI 1.6-4.8), and

For peer review only

lopinavir/ritonavir-based cART (aHR=3.7, 95% CI 1.0-13.3). The prevalence of lipodystrophy was 23.6%. Prior exposure to a stavudine-containing regimen was independently associated with lipodystrophy (aHR=3.1, 95% CI 1.6-6.1).

Conclusion

Our study revealed 38 % of the participants had MetS indicating considerable CVD risks. Independent risk factors for MetS were BMI ≥ 25 kg/m^2, efavirenz and lopinavir/ritonavir-based cART, female sex, and age ≥ 45 years. Besides prevention, CVD risk stratification and management will reduce morbidity & mortality in PWH.

Keywords: Metabolic Syndrome, HIV, cART, Ethiopian

ARTICLE SUMMARY

Strengths and Limitations of the Study

1. This study examined the cardiovascular risk factors and MetS among PWH on cART in a sub-Saharan African setting.
2. This study has the largest sample size compared with other studies done in Ethiopia about MetS and lipodystrophy in PWH on cART.
3. Complete nutritional assessment is lacking.
4. Advanced techniques for the assessment of lipodystrophy were not utilized.

For peer review only

INTRODUCTION

HIV/AIDS is a global pandemic. There were an estimated 38.4 million individuals living with HIV infection in 2021.[1] More than 95% of the world's population with HIV reside in low- and middle-income countries,[2] and two-thirds are in sub-Saharan Africa.[1] As the number of people receiving combination antiretroviral therapy (cART) progressed to 28.7 million in 2021,[1] AIDS-related mortality has decreased by half compared with 2010.[3] Such a phenomenal decrease in AIDS-related mortality is mainly attributed to the reduction of opportunistic infections.

People with HIV infection (PWH) receiving virus-suppressive cART will have immune reconstitution. Consequently, their life expectancy has become similar to HIV-uninfected individuals.[4] The Data collection of adverse events of Anti-HIV Drugs (D: A: D) study revealed that 289 of 2482 (11.6%) deaths were related to cardiovascular disease (CVD).[5] There is a shift from AIDS- related mortality to non-AIDS- related mortality as shown by a 2006 Antiretroviral therapy cohort collaboration(ART-CC). The study indicated that the frequency of opportunistic infection as a cause of death in PWH decreased from 32% to 19%. In contrast, mortality from CVD causes and type 2 diabetes mellitus complications increased from1.3% to 4.3%.[6,7] CVD has become a particular concern because of cART-induced metabolic changes, the high prevalence of cardiovascular risk factors in PWH, and growing evidence of HIV-accelerated inflammatory processes that promote atherosclerosis.[8] As a consequence of some antiretroviral drugs changing the body's morphology because of fat re-distribution and causing dyslipidemia, there is an increased risk for cardiovascular morbidity and mortality in PWH.[9] Metabolic syndrome (MetS), a combination of known cardiovascular risk factors, and HIV-associated lipodystrophy have been reported in different countries.[10,11] In PWH, metabolic

For peer review only

syndrome was detected in 21.1%, and HIV- associated lipodystrophy was detected in 12.1%.[12] Hypertriglyceredimeia prevalence was 15.2%.[13] Individual components of MetS and metabolic changes with lipodystrophy are known to be risk factors for CVD.

Identifying CVD risk factors in PWH on cART has public health significance. We evaluated the prevalence of MetS, Lipodystrophy, and related risk factors among Ethiopian PWH on cART at two tertiary-level hospitals in Addis Ababa, Ethiopia.

METHODS

STUDY DESIGN AND ELIGIBILITY

A cross-sectional study was conducted at tertiary referral hospitals, namely Tikur Anbessa Specialized Hospital (TASH) and Zewditu Memorial Hospital (ZMH), in Addis Ababa, Ethiopia. TASH is Addis Ababa University's main teaching hospital and the Addis Ababa Health Bureau administers ZMH. Addis Ababa has 2.7 million people accounting for nearly 4% of the Ethiopian population.[14] TASH and ZMH have a total of 4738 and 7200 HIV patients on cART at the time of the study respectively. In these institutions, the largest federal government-supported HIV/AIDS care and treatment program including the provision of cART and opportunistic infections prophylaxis and treatment is taking place. The study was conducted from July 2018- June 2019. The inclusion criteria were PWH, age 18 and above, on cART, and adherent to their treatment [Adherence to their treatment for ≥95% (≤ 3 doses missed out of 60 doses)] and taking cART for at least 12 months.[12,13]

.

For peer review only

The exclusion criteria were: non-adherence to cART, diagnosis of type 2 diabetes mellitus or hypertension before cART initiation, hypothyroidism, chronic renal failure, chronic corticosteroids use, oral contraceptives, Pregnancy, unable to undergo weight, height, and abdominal circumference measurements, unwilling to provide a blood sample for analysis and subjects who are too sick to be interviewed and examined.

The study subjects were screened by reviewing their health records and with interviews using structured questionnaires. A total of 518 subjects were recruited for the study using a simple random sampling method.

PATIENT AND PUBLIC INVOLVEMENT

There was no patient or public involvement in the study design, or conduct, or reporting, or dissemination plans of our study findings.

DATA COLLECTION AND QUALITY ASSURANCE

Data were collected by trained and qualified HIV specialty clinic nurses through interviews about sociodemographic characteristics such as age, sex, educational status, income, and occupation. The patient's health record was reviewed for CD4 counts and HIV RNA levels measured in the last 6 months before study enrollment. Data about the duration of HIV and the course of treatment with cART was examined. The data include the duration of treatment with the current and changed regimen, the specific regimens, regimen changes, the reason for the change, and the total duration of treatment with cART regimens was collected. The structured questionnaire also contained rest of clinical questions. The patient's self-report during interview and physical examination were used to collect body fat redistribution data of lipodystrophy manifestations including loss of buccal fat pad, central adiposity with/without extremity wasting,

For peer review only

dorsocervical/supraclavicular fat pad, and breast enlargement that were documented during the patients' interview and physical examination. The height and body weight were measured in centimeters and kilograms, respectively, using a digital balance with height measurement attached to it. The height measurements were converted into meters. Body mass index (BMI) was calculated as a ratio between the weight in kilograms and the square of the height in meters.[15] Abdominal waist circumference measurement was taken at the approximate midpoint between the lower margin of the last palpable rib and the top of the iliac crest as described in WHO guidelines.[16]

Using an automated sphygmomanometer, resting blood pressure in mmHg was measured based on standard definitions.[17] Morning Fasting venous blood sample, 5ml, was collected after an overnight, minimum of eight hours, fasting for fasting blood sugar (FBS), Total Cholestrol (TC), Triglyceride (TG), High Density Lipoprotien-Cholestrol (HDL-C), and Low Density Lipoprotien-Cholestrol (LDL-C) levels determination. Blood samples were collected in Gel and clot activator vacutainer tubes (Henso Medical (Hangzhou) Co.,Ltd.), taken to the lab in the same morning, kept at room temperature, and analyzed within the same day. Laboratory tests were made for FBS, TC, TG, HDL-C, and LDL-C by direct measurement using reagents (Human Diagnostics worldwide LiquiColor, Germany-Wiesbaden) for all by Mendray chemistry machine. Data were checked for accuracy, consistency, and cleanliness.

ASSESSMENT OF METABOLIC SYNDROME AND LIPODYSTROPHY

MetS was defined using International Diabetes Federation(IDF) and the National Cholesterol Education Program's Adult Treatment Panel (ATPIII) criteria.[18,19] IDF criteria are waist circumference ≥ 80 cm in women and ≥94 cm in men plus two of the following: triglycerides ≥ 150 mg/dl or drug treatment for high TG , HDL <40 mg/dl in men or <50 mg/dl in women or

For peer review only

drug treatment for low HDL-C, FBS ≥ 100 mg/dl or treatment for diagnosed diabetes, or blood pressure ≥130/85 mmHg or drug treatment for hypertension. ATPIII criteria are three of the following: waist circumference ≥88 cm in women and≥102 cm in men, triglycerides ≥ 150 mg/dl or drug treatment for elevated TG, HDL <40 mg/dl in men or <50 mg/dl in women or drug treatment for low HDL-C, FBS ≥ 100 mg/dl or drug treatment for elevated blood glucose, or blood pressure ≥130/85 mmHg or drug treatment for hypertension. Dyslipidemia was defined by ATP IIIcriteria with TC < 200 mg/dl as desirable, TG <150 mg/dl as normal, HDL-C < 40 mg/dl and <50 mg/ dl for men and women respectively as low, and LDL-C <100 mg/dl as optimal.[19] Lipodystrophy was defined as having any of clinical manifestations, self-reported or on physical examination, of loss of buccal fat pad, abdominal obesity with/without extremity wasting, breast enlargement, or dorsocervical or supraclavicular fat pad accumulation with or without signs of insulin resistance.[20-23]

STATISTICAL ANALYSIS

Data were entered into an Excel sheet, cleaned, and analyzed with SPSS statistical package version 25. Descriptive statistical analysis was made for sociodemographic and clinical variables and reported as frequency and percent, mean ± SD, or median + IQR. The prevalence of MetS and Lipodystrophy was computed and reported as percentages. Bivariate analysis was used to identify significant predictors of MetS and Lipodystrophy. Independent variables showing association during bivariate analysis were fed into multivariate models to control for confounding variables and analyzed using multivariable logistic regression. Because only a few study participants had the habit of smoking this factor was not tested for association with dependent variables. All significant test values were two-sided, and the significance level was set at $p < 0.05$.

For peer review only

RESULTS

PATIENTS CHARACTERISTICS

A total of 518 participants were enrolled, men [170 (32.8%)] and women [347 (67.0%)], and their mean (SD) age was 45.3 (10.7) years. Most of the study participants were private workers (44%) followed by civil servants (21%). Nearly 60% of the participants took a fixed-formulation tablet consisting of tenofovir, lamivudine, and efavirenz during the study.(Table. 1) In general, 213 (41.1%) of the participants had their first-line cART regimen subsequently changed. The changed regimens consisted of stavudine, lamuvidine, efavirenz or nevarapin [69(32.4%)]; and ziduvodine, lamuvidine, efavirenz or nevarapin [91(42.7%)].

The commonest reasons for the changes were HIV treatment failure [68(31.9%)], lipodystrophy [75(35.2%)], and anemia[26(12.2%)].The mean(SD) duration of HIV diagnosis, total duration of cART, treatment with current cART, and treatment with initially changed cART in years were 10.8(3.85), 9.97(3.63), 7.90(3.79), and 5.1(3.10) respectively. The mean BMI(SD) was 24.6(5.02) kg/m^2 and the mean abdominal waist circumference (SD) was 90.6(9.5) cm. The median CD4 cell counts of the study population was 460 (IQR:326.5,633.5) cells/mm^3.(Table.1)

For peer review only

Table 1: Sociodemographic and Clinical Characteristics of Study Participants at TASH* and ZMH*, Addis Ababa, Ethiopia

Sociodemographic and Clinical Variables	Frequency	Percent
**Sex**		
Male	170	32.8
Female	347	67.0
**Education**		
No formal Education attended	48	9.30
Primary and Secondary School	386	74.5
Diploma and higher Degree	84	16.2
**Occupation**		
Private works	227	43.8
Civil Servant	107	20.7
Merchant	28	5.40
Housewife	69	13.3
Miscellaneous others	87	16.8
**Types of current cART†**		
[1d,1c,1g,1h,1f]	158	30.5
1e	297	57.3
[2g,2h,2i,2f]	63	12.2
**Initial cART Changed**		
Yes	213	41.1
No	305	58.9
**Types of Changed Initial cART†**		
[1a,1b]	69	32.4
[1c,1d]	91	42.7
[1e,1g,1f]	32	15.0
[2i,2f,2h]	16	7.50
Missing-not traceable	05	2.40
**Reasons for change of cART‡**		
Treatment Failure	68	31.9
Lipodystrophy	75	35.2
Anemia	26	12.2
Others	30	14.1
Missing-not traceable	14	6.60
	**Mean ± SD**	**95% CI for Mean**
**Age**	45.3±10.74	44.37-46.20
**Duration Of:**		
HIV since diagnosed	10.8±3.85	10.46-11.12
Treatment with cART	9.97±3.63	9.65-10.27
Treatment with Current cART	7.90±3.79	7.51-08.19
Treatment with Changed cART	5.10±3.10	4.68-05.58

**BMI (Kg/m²)**	24.6±5.02	23.97-25.40
**Waist Circumference (cm)**	90.6±9.48	89.09-91.90
**Blood Pressure (mmHg)**		
Systolic	122±15.38	119.9-124.3
Diastolic	79.5±10.9	77.96-81.05
	**Median**	**IQR**
**Recent CD4 Count (cells/mm³)**	460	.[326.5, 633.5]

*TASH: Tikur Anbessa Speciaized Hospital, ZMH: Zewditu Memorial Hospital
†cART: combined Antiretroviral Therapy;**1a:** Stavudine (D4T)/ Lamuvidin (3TC) /Nevarapin(NVP); **1b:** D4T/3TC/ Efaverinez (EFV); **1c:** Zidovudine (ZDV)/3TC/ NVP; **1d:** ZDV/ 3TC/ EFV; **1e**: Tenofovir (TDF)/3TC/EFV; **1f:**TDF/3TC/NVP; **1g:**Abacavir(ABC)/3TC/EFV;**1h:** ABC/3TC/NVP; **2e:**AZT/3TC/ Ritonavir boosted Lopinavir (LPV/r); **2f:**AZT/3TC/ Ritonavir boosted Atazanavir (ATV/r)**; 2g:**TDF/3TC/ (LPV/r); **2h:**TDF/3TC/ ATV/r;**2i:** ABC/3TC/LPV/r
‡ Participants may have more than one reason for change of cART

PREVALENCE OF METABOLIC SYNDROME, LIPODYSTROPHY, HYPERTRIGLYCERIDEMIA, AND DISTRIBUTION OF LIPODYSTROPHY MANIFESTATIONS

In this cohort of 518 study participants, the prevalence of MetS by IDF 2006 criteria was found to be 193 (37.3%) and by ATPIII 2005 criteria 195(37.6%). The Prevalence of Lipodystrophy was 23.6% (122 cases). The prevalence of Hypertriglecridemia was 40.7% (211 cases).(Table 2) Central adiposity ±extremity wasting was the highest 80(15.4%) participants reported lipodystrophy manifestation followed by loss of buccal fat pad 76(14.7%). The highest lipodystrophy manifestation by physical exam was central adiposity ±extremity wasting 61(11.8%) followed by loss of buccal fat pad 60 (11.6%).(Table 2)

For peer review only

Table 2: Prevalence of metabolic syndrome, lipodystrophy, hypertriglyceridemia, and the distribution of lipodystrophy manifestations in HIV patients on cART* at TASH* and ZMH*, Addis Ababa, Ethiopia

Variables	Frequency	Percent
**Metabolic Syndrome IDF 2006**		
Yes	193	37.3
No	325	62.7
**Metabolic Syndrome ATP III 2005**		
Yes	195	37.6
No	323	62.4
**Lipodystrophy**		
**Participants reported**		
Yes	117	22.6
No	401	77.4
**Physical Exam**		
Yes	122	23.6
No	396	76.4
**Hypertrigleceridemia**		
Yes	211	40.7
No	307	59.3
**Lipodystrophy manifestations:**		
**Participants reported**		
**Loss of buccal fat Pad**		
Yes	76	14.7
No	442	85.3
**Central adiposity ±extremity wasting**		
Yes	80	15.4
No	438	84.6
**Supraclavicular/dorsocervical fat pad**		
Yes	39	7.5
No	479	92.5
**Breast Enlargement**		
Yes	31	6.0
No	486	93.8
**Lipodystrophy manifestations: Physical Examination**		
Loss of buccal fat pad	60	11.6
Central adiposity ±extremity wasting	61	11.8
Supraclavicular/dorsocervical fat pad	1	0.2
No Sign of Lipodystrophy	396	76.4

*cART: combined Antiretroviral Treatment, TASH: Tikur Anbessa Specialized Hospital, ZMH: Zewditu Memorial Hospital

ASSOCIATED RISK FACTORS FOR METABOLIC SYNDROME AND LIPODYSTROPHY

Bivariate regression analysis revealed a significant association with MetS between age ≥45 years [cHR=1.68, 95%CI = 1.1-2.5, p=0.009], female sex [cHR=1.71, 95%CI=1.2-2.5, p=0.003], BMI(kg/m^2) ≥ 25 [cHR=3.1, 95%CI = 2.1-4.4), p=0.001], and EFV based cART regimen [cHR = 2.8, 95%CI= 1.71-4.65, p=0.001] based on IDF 2006 criteria.

In contrast to the nevirapin (NVP) based-regimen, the lopinavir-based regimen had cHR = 2.8, 95% CI = 0.94-8.26. Independent risk factors for MetS with multivariable regression analysis were age ≥45 years [aHR=1.8, 95%CI= 1.2-2.4, p=0.006], female sex[aHR= 1.8, 95%CI= 1.1-2.8, p=0.013], BMI (kg/m^2) ≥ 25 [aHR= 2.71 , 95%CI= (1.8-4.1), p=0.001], EFV based cART regimen [aHR=2.8 95%CI = 1.6-4.8, p=0.001], and LPV based cART regimen [aHR= 3.7, 95%CI = 1.0-13.3), p=0.043]. CD4 count above 350 cells/mm^3 was not significantly associated with MetS in this study [cHR= 1.7, 95%CI = 0.8-3.6), p=0.143, aHR =1.5, 95%CI= 0.7-3.4%), p=0.320].(Table.3)

For peer review only

Table 3: Associated Risk Factors for Metabolic Syndrome among HIV patients on cART* at TASH* and ZMH*, Addis Ababa, Ethiopia

	Metabolic Syndrome †		cHR (95%CI)	P-Value	aHR (95%CI)	P-Value
**Variables**	Yes	No				
**Age (yrs)**						
**<45**	80(31.0%)	178(69.0%)	1		1	
**≥45**	113(43.5)	147(56.5%)	1.68 (1.14-2.49)	0.009	1.79(1.18 -2.37)	0.006
**Sex**						
**Male**	50 (29.4%)	120 (70.6%)	1		1	
**Female**	143(41.2%)	204 (58.8%)	1.71(1.19 -2.45)	0.003	1.79(1.13 - 2.83)	0.013
**Recent CD4 Count (cells/mm^3)**						
**<200**	11 (29.0%)	27 (71.0%)	1		1	
**200-350**	30 (29.4%)	72 (70.6%)	1.02 (0.45-2.32)	0.957	0.99(0.41-2.40)	0.989
**>350**	143(41.3%)	203(59.7%)	1.73(0.83-3.60)	0.143	1.50(0.67-3.35)	0.320
**BMI (kg/m^2)**						
**≤24.9**	83 (27.0%)	225(73.0%9	1		1	
**≥25**	108 (53.0%)	6 (47.0%)	3.05(2.10-4.43)	0.001	2.71(1.81-4.06)	0.001
**Current cART‡**						
**NVP based**	24(21.8%)	86(79.2%)	1		1	
**EFV based**	152(44.1%)	193(55.9%)	2.82(1.71-4.65)	0.001	2.79(1.62-4.80)	0.001
**LPV based**	7 (43.7%)	9 (56.3%)	2.79(0.94-8.26)	0.640	3.72(1.04-13.3)	0.043
**ATV based**	10 (21.3%)	37(78.7%)	0.97(0.42-2.23)	0.940	1.03(0.41-2.60)	0.954

* cART: combined Antiretroviral Treatment, TASH: Tikur Anbessa Specialized Hospital, ZMH: Zewditu Memorial Hospital

†Based on IDF 2006 Metabolic Syndrome Diagnostic Criteria

‡Current cART: NVP: Nevirapin, EFV:Efavirenz, LPV: Lopinavir, ATV: Atazanavir

Associated risk factors for Lipodystrophy on bivariate analysis were exposure to a stavudine containing regimen [cHR= 3.1, 95%CI= 1.7-5.7, p=0.001] and BMI(kg/m^2) ≥ 25 [cHR= 2.8, 95%CI= 1.8-4.2, p=0.001].. Age above 45 years was not found to be associated with lipodystrophy in our study(p=0.567). An independent risk factor for lipodytrophy was a cART regimen containing stavudin [aHR= 3.1, 95%CI= 1.6-6.1, p=0.001]. There was no significant

For peer review only - http://bmjopen.bmj.com/site/about/guidelines.xhtml

association between CD4 count and recent cART regimen and Lipodystrophy in this study.(Table 4)

Table 4: Associated Risk factors for Lipodystrophy among HIV Patients on cART* at TASH*and ZMH*, Addis Ababa, Ethiopia

	Lipodystrophy Sign(s)		cHR (95%CI)	*p*-value	aHR (95%CI)	*p*-Value
**Variables**	**Yes**	**No**				
**Initial cART†**						
**D4T Exposed**	36(52.2%)	33(47.8%)	3.09(1.68-5.68)	0.001	3.13(1.6-6.12)	0.001
**D4T Not exposed**	35(26.1%)	99(73.9%)	1		1	
**CD4 Count (cells/mm^3)**						
**<200**	6(16.7%)	30(83.3%)	1		1	
**200-350**	18(17.3%)	86(82.7%)	1.14(0.42-3.14)	0.795	0.63(0.18-2.18)	0.462
**>350**	92(26.6%)	254(73.4%)	1.93(0.78-4.77)	0.153	0.62(0.27-1.41)	0.254
**BMI* (kg/m^2)**						
**≤24.9**	71(34.7%)	133(65.2%)	1		1	
**≥25**	50(16.2%)	258(83.8%)	2.76(1.81-4.18)	0.001	1.48(0.76-2.86)	0.249
**Current cART‡**						
**NVP based**	24(21.8%)	86(78.2%)	1		1	
**EFV based**	85(24.6%)	260(75.4%)	1.17(0.70-1.96)	0.547	1.20(0.52-2.78)	0.666
**LPV based**	0	16(100%)	0.00(00-)	0.998	0.00(0.00-)	0.999
**ATV based**	13(27.7%)	34(72.3%)	1.37(0.63-3.00)	0.431	0.90(0.29-2.77)	0.857

*TASH: Tikur Anbessa Specialized Hospital, ZMH: Zewditu Memorial Hospital, BMI: Body Mass Index, cART: combined Antiretroviral Therapy
†Intial cART regimen: for those participants whose drugs were changed to the current cART regimen ; D4T : Stavudine
‡Current cART: NVP: Nevirapin, EFV:Efavirenz, LPV: Lopinavir, ATV: Atazanavir

DISCUSSION

Our study revealed the prevalence of MetS is 37.3% by IDF 2006 criteria and 37.6% by ATP III 2005 criteria.[18,19] In contrast to a MetS prevalence in an HIV-negative study population with hypertension in Northern Ethiopia by Tachebele et al.[24] [40.7% IDF and 39.3% ATPIII], our finding was slightly lower. The prevalence of MetS was higher with both criteria in our study than the 14% reported by Samaras and colleagues,[25] (males,72.8%) by IDF criteria and 18%

For peer review only

(males,84.9%) by ATPIII criteria. Berhane et al.[12] reported from Southern Ethiopia a prevalence of 21.1% by IDF criteria. The prevalence of MetS in our setting is higher than that reported in a Tanzanian study which revealed a 25.6% prevalence using IDF criteria.[26] This study's finding of the prevalence of MetS is lower than an Italian study that reported 45.4% prevalence by ATPIII criteria.[27] Compared with our study finding, the prevalence of MetS using IDF criteria was 4.8% (8.6% in females and 1.8% in males) in the national NCD STEPS survey in the Ethiopian general population.[28] Undoubtedly, our finding indicates a significantly higher risk of CVD among PWH receiving cART. The above discrepancies in the prevalence rates are partly explained by the time differences between the studies, the types of cART taken, and the populations studied. Even though the IDF criteria appears to be more stringent because of the requirement of waist circumference, it also allows more patients with lesser waist circumference to be included as was evident in this result indicating a closer prevalence to the ATP III criteria. Similarly, Samaras et al.[25] have found a higher prevalence of MetS by ATPIII criteria. A slightly higher value of MetS prevalence with IDF criteria was reported by Hirigo and Tesfaye in Southern Ethiopia, 24.3% and 17.8 % using the IDF and NCEP-ATP III criteria, respectively.[29]

Independent predictors of MetS in our study following adjustment for CD4 count were age above 45 years, female sex, BMI (kg/m^2) above 25, EFV-based cART regimen, and LPV-based cART regimen. Similar to our findings, studies in Ethiopia and Tanzania have found a significant association between female sex and MetS.[29,26] This is in line with other reports.[25,30] In contrast, one study revealed that gender has no significant association with MetS.[31] Similar to our study findings, Hirigo and Tesfaye have reported an increasing age above 40 years to be significantly associated with MetS.[29] This is in line with the report from Spain that has shown

For peer review only - http://bmjopen.bmj.com/site/about/guidelines.xhtml

an association between increasing age and MetS.[25] Similar to our study finding, higher BMI was significantly associated with MetS in other studies.[25,29] Furthermore, several other studies illustrated that the BMI is a quantitative predictor of MetS.[32-34]

Current use of EFV- based regimens is significantly associated with MetS in our study. This is supported by the fact that nonucleoside reverse transcriptase inhibitors impair lipid metabolism as a class,[35] and efavirenz leads to increased inflammation,[36,37] impaired adipogenesis and adipocyte differentiation,[36] impaired metabolism,[36,37] and no mitochondrial toxicity.[37] Efavirenz use is associated with increased apoptosis, increased mitochondrial mass, and oxidative stress in hepatic cells. Disruption in mitochondrial membrane potential was observed in hepatocytes promoting cytochrome c release and apoptosis.[38,39] Efavirenz also inhibits complex I of the electron transport chain (ETC) stimulating ROS production and a decrease in ATP which leads to an increase in the lipid content of hepatic cells.[40] Efavirenz also caused increased mitochondrial depolarization and altered mitochondrial morphology leading to mitophagy (clearance of damaged mitochondria) in neuronal cells.[41] Mitochondrial dysfunction promotes the progression of MetS by encouraging the occurrence of inflammation and oxidative stress. Efavirenz also acts as a pregnane X receptor agonist that promotes dyslipidemia and hypercholesteremia.[42] Non-nucleoside reverse transcriptase inhibitors (NNRTIs) promote MetS by reducing insulin sensitivity through their pro-inflammatory effects. NNRTIs are known for their reduction of adiponectin concentrations through increased expression of TNF-a, IL-6, and IL-1B. The reduction in the insulin sensitivity modulator, adiponectin, allows for the progression of insulin resistance.[43] Current EFV treatment, has been correlated with elevated markers of atherosclerosis.[44] The finding of liponavir- based cART regimen is in line with the independent association with MetS in a study in Barcelona,

For peer review only

Spain.[25] This is supported by the molecular mechanism of the drug that causes substantial impairment of adipocyte differentiation and lipid or glucose metabolism, pronounced decrease in mitochondrial proteins, and increased inflammation.[45]

The prevalence of lipodystrophy in this study was found to be 22.6% by subjective participants report and 23.6% by objective assessment for lipodystrophy manifestations. Our study finding is higher than the report of 12.1% by Berhane et al.[12] from South West Ethiopia and lower than the 68.3% prevalence in the study from Addis Ababa.[13] In our study, the higher prevalence compared to Berhane et al.[12] finding might be attributable to the larger sample size and the lower prevalence compared to Feleke et al.[13] finding may be explained by the trends of cART drug prescription that has been towards withdrawal of Stavudine. In our study, the prevalence of hypertriglyceridemia ≥ 150 mg/dl, a marker of lipodystrophy with insulin resistance and a component of MetS, was found in 40.7% of participants. This finding is closer to the finding of 43.5% prevalence in urban Tanzanian HIV population on cART,[26] higher than the 15.2% prevalence finding in Addis Ababa,[13] and lower than the report of 59.6% prevalence in a study in defense hospital in Addis Ababa.[46] Unadjusted, Lipodystrophy was significantly associated with a Stavudine- containing regimen and BMI (kg/m^2) ≥ 25. However, after adjustment for CD4 count (cells/mm^3) and current cART, only D4T exposure was significantly associated with Lipodystrophy. This finding of strong association between D4T and lipodystrophy is in line with previous reports.[47] This is because of the nucleoside reverse transcriptase class effect on impairing lipid metabolism,[35] and D4T effect of reduction of mitochondrial DNA.[48,49] Caron etal.[50] in their in vitro study have shown that Stavudin leads to decreases in lipid content, survival of adipose cells, and mitochondrial activity. The amount of mtDNA was found to be significantly decreased among patients receiving NRTIs indicating the mechanistic effect

For peer review only

of Stavudin on mitochondrial toxicity.[51] The resultant inflammatory cytokines such as Interleukins results in adipose cell destruction. This is known to impair Insulin signaling function and glucose transport. ART-associated lipodystrophy increases the risk for dyslipidemia, insulin resistance, diabetes mellitus, and heart disease.[52]

Limitations of this study are the cross sectional design, difficulty to trace some of the records on the initial cART regimen from old records, difficulty to get adequate samples for some of the variables that may have influenced the outcome like patients on Atazanavir based regimen, and fewer patients who were smokers or taking alcohol to identify their potential correlation in the study participants, and fewer patients on Liponavir based regimen as well. Besides, a complete nutritional assessment was not made. Advanced techniques for the assessment of lipodystrophy were not utilized because of availability. Of all the studies done in Ethiopia on MetS and Lipodystrophy, this is the one with the largest sample size and laboratory test results available for all participants.

CONCLUSION

Metabolic Syndrome was diagnosed in 37.3% and 37.6% of PWH using IDF and ATPIII criteria respectively. The higher prevalence of MetS is associated with female sex, age above 45 years, BMI above 25 Kg/m^2, EFV- based and Liponavir-based cART regimens. Hypertriglyceridemia was common among PWH receiving cART indicating cardiovascular and metabolic risks. Lipodystrophy was detected in 23.6% of study participants. Previous stavudine exposure is significantly associated with lipodystrophy. These metabolic changes among those on cART place them at high risk of cardiovascular events. Therefore, screening for MetS in PWH receiving cART and managing modifiable CV risk factors are highly recommended.

For peer review only

Furthermore, a prospective cohort study is recommended to evaluate MetS and lipodystrophy and the related CV outcomes among patients on cART.

AUTHOR CONTRIBUTION

AA, IO, and WA conceptualized the study. AA collected the primary data, conducted the analysis, and drafted the manuscript. HY, AR, AS, IO, and WA contributed to conceptualization, collection of data, and data analysis. AA, HY, AR, AS, IO, and WA have read, reviewed, and approved the article.

FUNDING

This collaborative research work is financially supported by an NIH funded project, Medical Education Partnership Initiative- Junior Faculty (D43TW010143).

ACKNOWLEDGMENT

The authors would like to acknowledge the study participants for their voluntary participation, the data collectors and the study centers staff at Zewditu Memorial Hospital and Tikur Anbessa Specialized Referral Hospital, for their invaluable support in the conduct of our study. In addition, the authors acknowledge Ms. Dorqa Woldesenbet (MPH) from School of Public Health-Addis Ababa University for her support in the statistical analysis.

CONFLICT OF INTEREST

The authors declare that this research was conducted in the absence of any commercial or financial conflict of interest.

For peer review only

ETHICAL CONSIDERATIONS

The study has been reviewed and approved by the Institution Review Board of the College of Health Sciences- Addis Ababa University with the Ref/ID# 077/17/IM and by Addis Ababa city regional health bureau Ethics review committee and have therefore been performed in accordance with the ethical standards of Declaration of Helsinki.

For peer review only

REFERENCES:

1. UNAIDS Global HIV & AIDS Statistics—2022 Fact Sheet. Available online: https://www.unaids.org/en/resources/fact-sheet
2. Kasper LD, Fauci SA, Hauser SL, Longo DL, Jameson LJ, Loscalzo; Harrison's Principles of Internal Medicine- 20th edition. 2018. New York. McGraw-Hill Medical Publishing Division.
3. IN DANGER: UNAIDS Global AIDS Update 2022. Geneva: Joint United Nations Programme on HIV/ AIDS; 2022. Licence: CC BY-NC-SA 3.0 IGO.
4. Obel N, Omland LH, Kronborg G *et al.* Impact of non-HIV and HIV risk factors on survival in HIV-infected patients on HAART: a population-based nationwide cohort study. *PLoS ONE*6, E22698 (2011).
5. Data Collection on Adverse Events of Anti H. I. V. drugs Study Group, Smith C, Sabin CA, Lundgren JD, etal. Factors associated with specific causes of death amongst HIV-positive individuals in the D:A:D study. AIDS 2010; 24: 1537–1548.
6. Smit C, Geskus R, walker S, et al. effective therapy has altered the spectrum of cause specific mortality following HIV seroconversion. AIDS. 2006;20:741-749
7. Samji H, Cescon A, Hogg RS, et al. Closing the Gap: increase in life expectancy among treated HIV positive individuals in the United States and Canada. PLoSone. 2013; 8(12):e81355

8. Lars G. Hemkens ,Heiner C. Bucher; novel therapeutic concepts, HIV infection and cardiovascular disease; European Heart journal , REVIEW; advance access published January 9,2014; doi:10.1093/euroheartj/eht528

9. Grover SA, Coupal L, Gilmore N, Mukherjee J. Impact of dyslipidemia associated with highly active antiretroviral therapy oncardiovascular risk and life expectancy. Am J Cardiol. 2005 Mar 1;95(5):586-91. This article on PubMed

10. Domingo P, Sambeat MA, Pérez A, Ordoñez J, Rodriguez J, Vázquez G. Fat distribution and metabolic abnormalities in HIV-infectedpatients on first combination antiretroviral therapy including stavudine or zidovudine: role of physical activity as a protective factor.AntivirTher. 2003; 8(3):223-231. This article on PubMed

11. Calza L, Manfredi R, Chiodo F. Dyslipidaemia associated with antiretroviral therapy in HIV-infected patients. J Antimicrob Chemother.2004;53(1):10-14. This article on PubMed

12. Tsegaye Berhane etal; prevalence of lipodystrophy and Metabolic syndrome among HIV positive individuals on HAART in Jimma, south west Ethiopia; Pan African Medical Journal. 2012; 13:43

13. Feleke Y, Fekade D, Mezgebu Y; prevalence of HAART associated metabolic abnormalities and lipodystrophy in HIV infected patients;Ethiopian medical Journal:2012, 50(3):221-230

14. Federal Democratic republic of Ethiopia, Ministry of Health: health sector development program IV 2010/11-2014/15.2010

15. Gibson RS. Principles of nutritional assessment. New York, Oxford University Press; 2005.15.

16. WHO. WHO STEPwise approach to surveillance (STEPS). Geneva: World Health Organization (WHO); 2008.

17. Perloff D, Grim C, Flack J et al. Human blood pressure determination by sphygmomanometry. Circulation. 1993;88:2460-2470.

18. International Diabetes Federation. The IDF consensus worldwide definition of the metabolic syndrome, 2006. file://www.idf.org/webdata/docs/MetS_def_update2006.pdf

For peer review only

19. Grundy SM, Brewer HB Jr, Cleeman JI, Smith SC Jr, Lenfant C; American Heart Association; National Heart, Lung, and Blood Institute. Definition of metabolic syndrome: Report of the National Heart, Lung, and Blood Institute/American Heart Association conference on scientific issues related to definition. Circulation. 2004; 109:433- 438. This article on PubMed

20. Freitas P, Santos AC, Carvalho Det al. Fat mass ratio: an objective tool to define lipodystrophy in HIV-infected patients under antiretroviral therapy. J Clin Densitom. 2010;13:197–203.

21. Bacchetti P, Gripshover B, Grunfeld C, et al. Fat distribution in men with HIV infection. J Acquir Immune Defic Syndr. 2005;40:121–31.

22. Jantarapakde J, Phanuphak N, Chaturawit C, et al. Prevalence of metabolic syndrome among antiretroviral-naive and antiretroviral-experienced HIV-1 infected Thaiadults. AIDS Patient Care STDS. 2014;28:331–40.

23. Carr A, Samaras K, Thorisdottir A, Kaufmann GR, Chisholm DJ, Cooper DA. Diagnosis, prediction, and natural course of HIV-1 protease-inhibitor associated lipodystrophy, hyperlipidaemia, and diabetes mellitus: a cohort study. Lancet. 1999;353:2093–9.

24. Belaynesh Tachebele, Molla Abebe, Zelalem Addis, and Nebiyu Mesfin; Metabolic syndrome among hypertensive patients at University of Gondar Hospital, North West Ethiopia: a cross sectional study; BMC Cardiovascular Disorders 2014, 14:177

25. Samaras K, Wand H, Law M, Emery S, Cooper D, Carr A. Prevalence of metabolic syndrome in HIV-infected patients receiving highly active antiretroviral therapy using international diabetes foundation and adult treatment panel III criteria. Diabetes Care. 2007;30:113–9.

26. Kagaruki G, Kimaro G, Mweya C etal.; Prevalence and Risk Factors of Metabolic Syndrome among Individuals Living with HIV and Receiving Antiretroviral Treatment in Tanzania; British Journal of Medicine & Medical Research 5(10): 1317-1327, 2015, Article no.BJMMR.2015.150 ISSN: 2231-0614

27. Gazzaruso C, Sacchi P, Garzaniti A, Fratino P, Bruno R, Filice G: Prevalence of metabolic syndrome among HIV patients. Diabetes Care 2002, 25:1253–1254.

For peer review only

28. Gebreyes Y, Goshu D, Geleta T etal; Prevalence of high bloodpressure, hyperglycemia, dyslipidemia, metabolic syndrome and their determinants in Ethiopia: Evidences from the National NCDs STEPS Survey, 2015 PLOS ONE | https://doi.org/10.1371/journal.pone.0194819 May 9, 2018

29. Agete Tadewos Hirigo, Demo Yemane Tesfaye; Influences of gender in metabolic syndrome and its components among people living with HIV virus using antiretroviral treatment in Hawassa, southern Ethiopia; *BMC Res Notes (2016) 9:145* DOI 10.1186/s13104-016-1953-2

30. Ivana K, Ariana V, Branka S, Mladen S, Josipa K, Silvije V. Metabolic syndrome in a metapopulation of Croatian island isolates. Croat Med J. 2006;47:585–92.

31. Lauda LG, Mariath AB, Grillo LP. Metabolic syndrome and its components in HIV-infected individuals. Rev Assoc Med Bras. 2011;57:178–81.

32. Jacobson DL, Tang AM, Spiegelman D, etal. Incidence of metabolic syndrome in a cohort of HIVinfected adults and prevalence relative to the US population (National Health and Nutrition Examination Survey). J Acquir Immune Defic Syndr. 2006;43:458–66.

33. Jericó C, Knobel H, Montero M, et al. Metabolic syndrome among HIV-infected patients: prevalence, characteristics, and related factors. Diabetes Care. 2005;28(132):137.

34. Mondy K, Overton ET, Grubb J etal.. Metabolic syndrome in HIV-infected patients from an urban, midwestern US outpatient population. Clin Infect Dis. 2007;44:726–73.

35. Minami R, Yamamoto M, Takahama S, Ando H, Miyamura T, Suematsu E. Comparison of the infl uence of four classes of HIV antiretrovirals on adipogenic diff erentiation: the minimal eff ect of raltegravir and atazanavir. *J Infect Chemother* 2011; 17: 183–88.

36. Diaz-Delfi n J, Domingo P, Mateo MG, et al. Effects of rilpivirine on human adipocyte diff erentiation, gene expression, and release of adipokines and cytokines. *Antimicrob Agents Chemother* 2012;56: 3369–75.

For peer review only - http://bmjopen.bmj.com/site/about/guidelines.xhtml

37. Diaz-Delfi n J, del Mar Gutierrez M, Gallego-Escuredo JM, et al. Eff ects of nevirapine and efavirenz on human adipocyte diff erentiation, gene expression, and release of adipokines andcytokines. *Antiviral Res* 2011; 91: 112–19.

38. Jamaluddin M.S., Lin P.H., Yao Q., Chen C. Non-nucleoside reverse transcriptase inhibitor efavirenz increases monolayer permeability of human coronary artery endothelial cells. *Atherosclerosis.* 2010;208:104–111. doi: 10.1016/j.atherosclerosis.2009.07.029.

39. Ganta K.K., Mandal A., Chaubey B. Depolarization of mitochondrial membrane potential is the initial event in non-nucleoside reverse transcriptase inhibitor efavirenz induced cytotoxicity. *Cell Biol. Toxicol.* 2017;33:69–82. doi: 10.1007/s10565-016-9362-9.

40. Blas-García A., Apostolova N., Ballesteros D., Monleon D., Morales J.M., Rocha M., Victor V.M., Esplugues J.V. Inhibition of mitochondrial function by efavirenz increases lipid content in hepatic cells. *Hepatology.* 2010;52:115–125. doi: 10.1002/hep.23647.

41. Purnell P.R., Fox H.S. Efavirenz induces neuronal autophagy and mitochondrial alterations. *J. Pharmacol. Exp. Ther.* 2014;351:250–258. doi: 10.1124/jpet.114.217869.

42. Gwag T., Meng Z., Sui Y., Helsley R.N., Park S.-H., Wang S., Greenberg R.N., Zhou C. Non-nucleoside reverse transcriptase inhibitor efavirenz activates PXR to induce hypercholesterolemia and hepatic steatosis. *J. Hepatol.* 2019;70:930–940. doi: 10.1016/j.jhep.2018.12.038.

43. Lagathu C., Kim M., Maachi M., Vigouroux C., Cervera P., Capeau J., Caron M., Bastard J.-P. HIV antiretroviral treatment alters adipokine expression and insulin sensitivity of adipose tissue in vitro and in vivo. *Biochimie.* 2005;87:65–71. doi: 10.1016/j.biochi.2004.12.007.

44. Rudolph L. Gleason Jr., Alexander W. Cualk, Daniel Seifu, etal; Current Efavirenz (EFV) or Ritonavir-Boosted Lopinavir (LPV/r) Use Correlates with Elevate Markers of Atherosclerosis in HIV-Infected Subjects in Addis Ababa, Ethiopia; PLOS ONE | DOI:10.1371/journal.pone.0117125 April 27, 2015

For peer review only

45. Capel E, Auclair M, Caron-Debarle M, Capeau J. Effects of ritonavirboosteddarunavir, atazanavir and lopinavir on adipose functionsand insulin sensitivity in murine and human adipocytes.*Antivir Ther* 2012; 17: 549–56.

46. Bayenes H, Ahmed M, Shenkute T, etal.;Prevalence and predictors of dyslipidemia on HAART and HAART naive HIV positive persons in Defense Hospital, Addis Ababa, Ethiopia; American Journal of Health Research.2014; 2(5): 303-309

47. Finkelstein JL, Gala P, Rochford R Glesby MJ and MehtaS.HIV/AIDS and lipodystrophy: Implications for clinical management in resource-limited settings;Journalof the International AIDS Society 2015, 18:19033http://www.jiasociety.org/index.php/jias/article/view/19033 | http://dx.doi.org/10.7448/IAS.18.1.19033

48. Feeney ER, van Vonderen MG, Wit F, et al. Zidovudine/lamivudine but not nevirapine in combination with lopinavir/ritonavir decreases subcutaneous adipose tissue mitochondrial DNA. *AIDS* 2012; 26: 2165–74.

49. McComsey GA, Daar ES, O'Riordan M, et al. Changes in fat mitochondrial DNA and function in subjects randomized to abacavir-lamivudine or tenofovir DF-emtricitabine with atazanavirritonavir or efavirenz: AIDS Clinical Trials Group study A5224s, substudy of A5202. *J Infect Dis* 2013; 207: 604–11.

50. Caron M, Auclair M, Lagathu C, et al. The HIV-1 nucleoside reverse transcriptase inhibitors stavudine and zidovudine alter adipocyte functions in vitro. AIDS. 2004;18(16):2127–2136. doi:10.1097/ 00002030-200411050-00004

51. Nolan D, Hammond E, Martin A, et al. Mitochondrial DNA depletion and morphologic changes in adipocytes associated with nucleoside reverse transcriptase inhibitor therapy. AIDS. 2003;17(9):1329–1338. doi:10.1097/00002030-200306130-00007

52. Fiorenza CG, Chou SH, Mantzoros CS. Lipodystrophy: pathophysiology and advances in treatment. Nat Rev Endocrinol. 2011;7 (3):137–150. doi:10.1038/nrendo.2010.199

For peer review only

For peer review only

Reporting checklist for cross sectional study.

Based on the STROBE cross sectional guidelines.

Instructions to authors

Complete this checklist by entering the page numbers from your manuscript where readers will find each of the items listed below.

Your article may not currently address all the items on the checklist. Please modify your text to include the missing information. If you are certain that an item does not apply, please write "n/a" and provide a short explanation.

Upload your completed checklist as an extra file when you submit to a journal.

In your methods section, say that you used the STROBE cross sectionalreporting guidelines, and cite them as:

von Elm E, Altman DG, Egger M, Pocock SJ, Gotzsche PC, Vandenbroucke JP. The Strengthening the Reporting of Observational Studies in Epidemiology (STROBE) Statement: guidelines for reporting observational studies.

		Reporting Item	Page Number
Title and abstract			
Title	#1a	Indicate the study's design with a commonly used term in the title or the abstract	2
Abstract	#1b	Provide in the abstract an informative and balanced summary of what was done and what was found	2
Introduction			
Background / rationale	#2	Explain the scientific background and rationale for the investigation being reported	4
Objectives	#3	State specific objectives, including any prespecified hypotheses	5
Methods			
Study design	#4	Present key elements of study design early in the paper	6
Setting	#5	Describe the setting, locations, and relevant dates, including periods of recruitment, exposure, follow-up, and data collection	6
Eligibility criteria	#6a	Give the eligibility criteria, and the sources and methods of selection of participants.	6

For peer review only

	#7	Clearly define all outcomes, exposures, predictors, potential confounders, and effect modifiers. Give diagnostic criteria, if applicable	8
Data sources / measurement	#8	For each variable of interest give sources of data and details of methods of assessment (measurement). Describe comparability of assessment methods if there is more than one group. Give information separately for for exposed and unexposed groups if applicable.	6,7,8
Bias	#9	Describe any efforts to address potential sources of bias	7
Study size	#10	Explain how the study size was arrived at	7
Quantitative variables	#11	Explain how quantitative variables were handled in the analyses. If applicable, describe which groupings were chosen, and why	9
Statistical methods	#12a	Describe all statistical methods, including those used to control for confounding	9
Statistical methods	#12b	Describe any methods used to examine subgroups and interactions	9
Statistical methods	#12c	Explain how missing data were addressed	n/a
Statistical methods	#12d	If applicable, describe analytical methods taking account of sampling strategy	n/a
Statistical methods	#12e	Describe any sensitivity analyses	n/a
Results			
Participants	#13a	Report numbers of individuals at each stage of study—eg numbers potentially eligible, examined for eligibility, confirmed eligible, included in the study, completing follow-up, and analysed. Give information separately for for exposed and unexposed groups if applicable.	10
Participants	#13b	Give reasons for non-participation at each stage	n/a
Participants	#13c	Consider use of a flow diagram	n/a
Descriptive data	#14a	Give characteristics of study participants (eg demographic, clinical, social) and information on exposures and potential confounders. Give information separately for exposed and unexposed groups if applicable.	10, 11
Descriptive data	#14b	Indicate number of participants with missing data for each variable of interest	11
Outcome data	#15	Report numbers of outcome events or summary measures. Give information separately for exposed and	12,13,14

For peer review only

		unexposed groups if applicable.	
Main results	#16a	Give unadjusted estimates and, if applicable, confounder-adjusted estimates and their precision (eg, 95% confidence interval). Make clear which confounders were adjusted for and why they were included	14,15,16
Main results	#16b	Report category boundaries when continuous variables were categorized	n/a
Main results	#16c	If relevant, consider translating estimates of relative risk into absolute risk for a meaningful time period	n/a
Other analyses	#17	Report other analyses done—e.g., analyses of subgroups and interactions, and sensitivity analyses	n/a
Discussion			
Key results	#18	Summarise key results with reference to study objectives	17,18,19
Limitations	#19	Discuss limitations of the study, taking into account sources of potential bias or imprecision. Discuss both direction and magnitude of any potential bias.	19
Interpretation	#20	Give a cautious overall interpretation considering objectives, limitations, multiplicity of analyses, results from similar studies, and other relevant evidence.	20
Generalisability	#21	Discuss the generalisability (external validity) of the study results	20
Other Information			
Funding	#22	Give the source of funding and the role of the funders for the present study and, if applicable, for the original study on which the present article is based	20

None The STROBE checklist is distributed under the terms of the Creative Commons Attribution License CC-BY. This checklist can be completed online using https://www.goodreports.org/, a tool made by the EQUATOR Network in collaboration with Penelope.ai

For peer review only

BMJ Open

THE PREVALENCE AND RISK FACTORS OF METABOLIC SYNDROME IN ETHIOPIA: DESCRIBING AN EMERGING OUTBREAK IN HIV CLINICS OF THE SUB-SAHARAN AFRICA. A CROSS SECTIONAL STUDY

Journal:	*BMJ Open*
Manuscript ID	bmjopen-2022-069637.R2
Article Type:	Original research
Date Submitted by the Author:	25-Sep-2023
Complete List of Authors:	Abdela, Abdurezak; Addis Ababa University, Department of Internal Medicine<br>Yifter, Helen; Addis Ababa University, Department of Internal Medicine<br>Reja, Ahmed; Addis Ababa University, Department of Internal Medicine<br>Shewaamare, Aster; Zewditu Memorial Hospital<br>Ofotokun, Ighovwerha; Emory University, Department of Internal Medicine<br>Degu, Wondwossen; Addis Ababa University, Department of Internal Medicine
**Primary Subject Heading**:	Diabetes and endocrinology
Secondary Subject Heading:	HIV/AIDS
Keywords:	HIV & AIDS < INFECTIOUS DISEASES, Lipid disorders < DIABETES & ENDOCRINOLOGY, Diabetes & endocrinology < INTERNAL MEDICINE

SCHOLARONE™ Manuscripts

I, the Submitting Author has the right to grant and does grant on behalf of all authors of the Work (as defined in the below author licence), an exclusive licence and/or a non-exclusive licence for contributions from authors who are: i) UK Crown employees; ii) where BMJ has agreed a CC-BY licence shall apply, and/or iii) in accordance with the terms applicable for US Federal Government officers or employees acting as part of their official duties; on a worldwide, perpetual, irrevocable, royalty-free basis to BMJ Publishing Group Ltd ("BMJ") its licensees and where the relevant Journal is co-owned by BMJ to the co-owners of the Journal, to publish the Work in this journal and any other BMJ products and to exploit all rights, as set out in our licence.

The Submitting Author accepts and understands that any supply made under these terms is made by BMJ to the Submitting Author unless you are acting as an employee on behalf of your employer or a postgraduate student of an affiliated institution which is paying any applicable article publishing charge ("APC") for Open Access articles. Where the Submitting Author wishes to make the Work available on an Open Access basis (and intends to pay the relevant APC), the terms of reuse of such Open Access shall be governed by a Creative Commons licence – details of these licences and which Creative Commons licence will apply to this Work are set out in our licence referred to above.

Other than as permitted in any relevant BMJ Author's Self Archiving Policies, I confirm this Work has not been accepted for publication elsewhere, is not being considered for publication elsewhere and does not duplicate material already published. I confirm all authors consent to publication of this Work and authorise the granting of this licence.

For peer review only

THE PREVALENCE AND RISK FACTORS OF METABOLIC SYNDROME IN ETHIOPIA: DESCRIBING AN EMERGING OUTBREAK IN HIV CLINICS OF THE SUB-SAHARAN AFRICA. A CROSS SECTIONAL STUDY

Word count = 3979

Authors: Abdurezak Ahmed[1], Helen Yifter[1], Ahmed Reja[1], Aster Shewaamare[2], Ighovwerha Ofotokun[3] , Wondwossen Amogne[4]

Corresponding author: Wondwossen Amogne, emial: wonamogne@yahoo.com, wondwossen.amogne @aau.edu.et, Department of Internal Medicine, Addis Ababa University, P.O.Box 2380 Addis Ababa-Ethiopia

[1] Endocrine Unit, Department of Internal Medicine, Addis Ababa University, Ethiopia. Emails in order of authors: abdurezakahmed1977@gmail.com, helenefbr@gmail.com, ahmedreja@yahoo.com
[2] HIV clinic, Zewditu Memorial Hospital, Addis Ababa, Ethiopia. Email: aster.shewaamare@gmail.com
[3]Division of Infectious Diseases, Department of Internal Medicine, Emory University, USA. Email: iofotok@emory.edu
[4] Division of Infectious Diseases, Department of Internal Medicine, Addis Ababa University, Ethiopia. Email:wonamogne@yahoo.com, wondwossen@aau.edu.et

For peer review only

ABSTRACT

Objectives

HIV-induced chronic inflammation, immune activation, and combination antiretroviral therapy (cART) are linked with adverse metabolic changes known to cause cardiovascular adversities. This study evaluates the prevalence of lipodystrophy, and metabolic syndrome (MetS), and analyzes risk factors in HIV-infected Ethiopians taking cART.

Methods

A multicenter cross-sectional study was conducted at tertiary-level hospitals. Eligible participants attending the HIV clinics were enrolled. Sociodemographic, anthropometric, clinical, HIV treatment variables, lipid profile, fasting blood glucose level, risk factors, and components of MetS, also lipodystrophy were studied. Data were analyzed by SPSS statistical package version 25 with descriptive and analytical statistics. For multivariable analysis of risk factors, a logistic regression model was used. Results were presented in frequency and percentages, Mean± Standard Deviation, or Median + interquartile ranges. Statistical significance was taken as $p<0.05$.

Results

Among 518 studied participants, two-thirds were females, and the mean age of the study population was 45 years (SD=11). The mean duration of cART was 10 years(SD=4). Median CD4 count was 460 cells/mm^3. The prevalence of MetS according to the Adult Treatment Panel III (2005) criteria was 37.6%. In multivariable analysis, independent risk factors for MetS were age ≥ 45 years (aHR= 1.8, 95% CI 1.2-2.4), female sex (aHR=1.8, 95% CI 1.1-2.8), BMI≥ 25 Kg/m^2 (aHR=2.7, 95% CI 1.8-4.1), efavirenz-based cART (aHR=2.8, 95% CI 1.6-4.8), and

For peer review only

lopinavir/ritonavir-based cART (aHR=3.7, 95% CI 1.0-13.3). The prevalence of lipodystrophy was 23.6%. Prior exposure to a stavudine-containing regimen was independently associated with lipodystrophy (aHR=3.1, 95% CI 1.6-6.1).

Conclusion

Our study revealed 38 % of the participants had MetS indicating considerable CVD risks. Independent risk factors for MetS were BMI ≥ 25 kg/m^2, efavirenz and lopinavir/ritonavir-based cART, female sex, and age ≥ 45 years. Besides prevention, CVD risk stratification and management will reduce morbidity & mortality in PWH.

Keywords: Metabolic Syndrome, HIV, cART, Ethiopian

ARTICLE SUMMARY

Strengths and Limitations of the Study

1. This study examined the cardiovascular risk factors and MetS among PWH on cART in a sub-Saharan African setting.
2. This study has the largest sample size compared with other studies done in Ethiopia about MetS and lipodystrophy in PWH on cART.
3. Complete nutritional assessment is lacking.
4. Advanced techniques for the assessment of lipodystrophy were not utilized.

For peer review only

INTRODUCTION

HIV/AIDS is a global pandemic. There were an estimated 38.4 million individuals living with HIV infection in 2021.[1] More than 95% of the world's population with HIV reside in low- and middle-income countries,[2] and two-thirds are in sub-Saharan Africa.[1] As the number of people receiving combination antiretroviral therapy (cART) progressed to 28.7 million in 2021,[1] AIDS-related mortality has decreased by half compared with 2010.[3] Such a phenomenal decrease in AIDS-related mortality is mainly attributed to the reduction of opportunistic infections.

People with HIV infection (PWH) receiving virus-suppressive cART will have immune reconstitution. Consequently, their life expectancy has become similar to HIV-uninfected individuals.[4] The Data collection of adverse events of Anti-HIV Drugs (D: A: D) study revealed that 289 of 2482 (11.6%) deaths were related to cardiovascular disease (CVD).[5] Though this analysis was made for data until 2008, DAD data were obtained in cohorts I-III in the period of December 1999-2001, then throughout spring of 2004, and until 2010 respectively.

There is a shift from AIDS- related mortality to non-AIDS- related mortality as shown by a 2006 Antiretroviral therapy cohort collaboration(ART-CC). The study indicated that the frequency of opportunistic infection as a cause of death in PWH decreased from 32% to 19%. In contrast, mortality from CVD causes and type 2 diabetes mellitus complications increased from1.3% to 4.3%.[6,7] CVD has become a particular concern because of cART-induced metabolic changes, the high prevalence of cardiovascular risk factors in PWH, and growing evidence of HIV-accelerated inflammatory processes that promote atherosclerosis.[8] As a consequence of some antiretroviral drugs changing the body's morphology because of fat re-distribution and causing dyslipidemia, there is an increased risk for cardiovascular morbidity and mortality in PWH.[9]

For peer review only

Metabolic syndrome (MetS), a combination of known cardiovascular risk factors, and HIV-associated lipodystrophy have been reported in different countries.[10,11] In PWH, metabolic syndrome was detected in 21.1%, and HIV- associated lipodystrophy was detected in 12.1%.[12] Hypertriglyceredimeia prevalence was 15.2%.[13] Individual components of MetS and metabolic changes with lipodystrophy are known to be risk factors for CVD.

Identifying CVD risk factors in PWH on cART has public health significance. We evaluated the prevalence of MetS, Lipodystrophy, and related risk factors among Ethiopian PWH on cART at two tertiary-level hospitals in Addis Ababa, Ethiopia.

METHODS

STUDY DESIGN AND ELIGIBILITY

A cross-sectional study was conducted at tertiary referral hospitals, namely Tikur Anbessa Specialized Hospital (TASH) and Zewditu Memorial Hospital (ZMH), in Addis Ababa, Ethiopia. TASH is Addis Ababa University's main teaching hospital and the Addis Ababa Health Bureau administers ZMH. Addis Ababa has 2.7 million people accounting for nearly 4% of the Ethiopian population.[14] TASH and ZMH have a total of 4738 and 7200 HIV patients on cART at the time of the study respectively. In these institutions, the largest federal government-supported HIV/AIDS care and treatment program including the provision of cART and opportunistic infections prophylaxis and treatment is taking place. The study was conducted from July 2018- June 2019. The inclusion criteria were PWH, age 18 and above, on cART, and adherent to their treatment [Adherence to their treatment for ≥95% (≤ 3 doses missed out of 60 doses)] and taking cART for at least 12 months.[12,13]

For peer review only

.

The exclusion criteria were: non-adherence to cART, diagnosis of type 2 diabetes mellitus or hypertension before cART initiation, hypothyroidism, chronic renal failure, chronic corticosteroids use, oral contraceptives, Pregnancy, unable to undergo weight, height, and abdominal circumference measurements, unwilling to provide a blood sample for analysis and subjects who are too sick to be interviewed and examined.

The study subjects were screened by reviewing their health records and with interviews using structured questionnaires. A total of 518 subjects were recruited for the study using a simple random sampling method.

PATIENT AND PUBLIC INVOLVEMENT

There was no patient or public involvement in the study design, or conduct, or reporting, or dissemination plans of our study findings.

DATA COLLECTION AND QUALITY ASSURANCE

Data were collected by trained and qualified HIV specialty clinic nurses through interviews about sociodemographic characteristics such as age, sex, educational status, income, and occupation. The patient's health record was reviewed for CD4 counts and HIV RNA levels measured in the last 6 months before study enrollment. Data about the duration of HIV and the course of treatment with cART was examined. The data include the duration of treatment with the current and changed regimen, the specific regimens, regimen changes, the reason for the change, and the total duration of treatment with cART regimens was collected. The structured questionnaire also contained rest of clinical questions. The patient's self-report during interview

For peer review only

and physical examination were used to collect body fat redistribution data of lipodystrophy manifestations including loss of buccal fat pad, central adiposity with/without extremity wasting, dorsocervical/supraclavicular fat pad, and breast enlargement that were documented during the patients' interview and physical examination. The height and body weight were measured in centimeters and kilograms, respectively, using a digital balance with height measurement attached to it. The height measurements were converted into meters. Body mass index (BMI) was calculated as a ratio between the weight in kilograms and the square of the height in meters.[15] Abdominal waist circumference measurement was taken at the approximate midpoint between the lower margin of the last palpable rib and the top of the iliac crest as described in WHO guidelines.[16]

Using an automated sphygmomanometer, resting blood pressure in mmHg was measured based on standard definitions.[17] Morning Fasting venous blood sample, 5ml, was collected after an overnight, minimum of eight hours, fasting for fasting blood sugar (FBS), Total Cholestrol (TC), Triglyceride (TG), High Density Lipoprotien-Cholestrol (HDL-C), and Low Density Lipoprotien-Cholestrol (LDL-C) levels determination. Blood samples were collected in Gel and clot activator vacutainer tubes (Henso Medical (Hangzhou) Co.,Ltd.), taken to the lab in the same morning, kept at room temperature, and analyzed within the same day. Laboratory tests were made for FBS, TC, TG, HDL-C, and LDL-C by direct measurement using reagents (Human Diagnostics worldwide LiquiColor, Germany-Wiesbaden) for all by Mendray chemistry machine. Data were checked for accuracy, consistency, and cleanliness.

ASSESSMENT OF METABOLIC SYNDROME AND LIPODYSTROPHY

MetS was defined using International Diabetes Federation(IDF) and the National Cholesterol Education Program's Adult Treatment Panel (ATPIII) criteria.[18,19] IDF criteria are waist

For peer review only

circumference ≥ 80 cm in women and ≥94 cm in men plus two of the following: triglycerides ≥ 150 mg/dl or drug treatment for high TG , HDL <40 mg/dl in men or <50 mg/dl in women or drug treatment for low HDL-C, FBS ≥ 100 mg/dl or treatment for diagnosed diabetes, or blood pressure ≥130/85 mmHg or drug treatment for hypertension. ATPIII criteria are three of the following: waist circumference ≥88 cm in women and≥102 cm in men, triglycerides ≥ 150 mg/dl or drug treatment for elevated TG, HDL <40 mg/dl in men or <50 mg/dl in women or drug treatment for low HDL-C, FBS ≥ 100 mg/dl or drug treatment for elevated blood glucose, or blood pressure ≥130/85 mmHg or drug treatment for hypertension. Dyslipidemia was defined by ATP IIIcriteria with TC < 200 mg/dl as desirable, TG <150 mg/dl as normal, HDL-C < 40 mg/dl and <50 mg/ dl for men and women respectively as low, and LDL-C <100 mg/dl as optimal.[19] Lipodystrophy was defined as having any of clinical manifestations, self-reported or on physical examination, of loss of buccal fat pad, abdominal obesity with/without extremity wasting, breast enlargement, or dorsocervical or supraclavicular fat pad accumulation with or without signs of insulin resistance.[20-23]

For peer review only

STATISTICAL ANALYSIS

Data were entered into an Excel sheet, cleaned, and analyzed with SPSS statistical package version 25. Descriptive statistical analysis was made for sociodemographic and clinical variables and reported as frequency and percent, mean ± SD, or median + IQR. The prevalence of MetS and Lipodystrophy was computed and reported as percentages. Bivariate analysis was used to identify significant predictors of MetS and Lipodystrophy. Independent variables showing association during bivariate analysis were fed into multivariate models to control for confounding variables and analyzed using multivariable logistic regression. Because only a few study participants had the habit of smoking this factor was not tested for association with

dependent variables. All significant test values were two-sided, and the significance level was set at $p < 0.05$.

RESULTS

PATIENTS CHARACTERISTICS

A total of 518 participants were enrolled, men [170 (32.8%)] and women [347 (67.0%)], and their mean (SD) age was 45.3 (10.7) years. Most of the study participants were private workers (44%) followed by civil servants (21%). Nearly 60% of the participants took a fixed-formulation tablet consisting of tenofovir, lamivudine, and efavirenz during the study.(Table. 1) In general, 213 (41.1%) of the participants had their first-line cART regimen subsequently changed. The changed regimens consisted of stavudine, lamuvidine, efavirenz or nevarapin [69(32.4%)]; and ziduvodine, lamuvidine, efavirenz or nevarapin [91(42.7%)].

The commonest reasons for the changes were HIV treatment failure [68(31.9%)], lipodystrophy [75(35.2%)], and anemia[26(12.2%)].The mean(SD) duration of HIV diagnosis, total duration of cART, treatment with current cART, and treatment with initially changed cART in years were 10.8(3.85), 9.97(3.63), 7.90(3.79), and 5.1(3.10) respectively. The mean BMI(SD) was 24.6(5.02) kg/m^2 and the mean abdominal waist circumference (SD) was 90.6(9.5) cm. The median CD4 cell counts of the study population was 460 (IQR:326.5,633.5) cells/mm^3.(Table.1)

For peer review only - http://bmjopen.bmj.com/site/about/guidelines.xhtml

Table 1: Sociodemographic and Clinical Characteristics of Study Participants at TASH* and ZMH*, Addis Ababa, Ethiopia

Sociodemographic and Clinical Variables	Frequency	Percent
**Sex**		
Male	170	32.8
Female	347	67.0
**Education**		
No formal Education attended	48	9.30
Primary and Secondary School	386	74.5
Diploma and higher Degree	84	16.2
**Occupation**		
Private works	227	43.8
Civil Servant	107	20.7
Merchant	28	5.40
Housewife	69	13.3
Miscellaneous others	87	16.8
**Types of current cART†**		
[1d,1c,1g,1h,1f]	158	30.5
1e	297	57.3
[2g,2h,2i,2f]	63	12.2
**Initial cART Changed**		
Yes	213	41.1
No	305	58.9
**Types of Changed Initial cART†**		
[1a,1b]	69	32.4
[1c,1d]	91	42.7
[1e,1g,1f]	32	15.0
[2i,2f,2h]	16	7.50
Missing-not traceable	05	2.40
**Reasons for change of cART‡**		
Treatment Failure	68	31.9
Lipodystrophy	75	35.2
Anemia	26	12.2
Others	30	14.1
Missing-not traceable	14	6.60
	**Mean ± SD**	**95% CI for Mean**
**Age**	45.3±10.74	44.37-46.20
**Duration Of:**		
HIV since diagnosed	10.8±3.85	10.46-11.12

Treatment with cART	9.97±3.63	9.65-10.27
Treatment with Current cART	7.90±3.79	7.51-08.19
Treatment with Changed cART	5.10±3.10	4.68-05.58
**BMI (Kg/m²)**	24.6±5.02	23.97-25.40
**Waist Circumference (cm)**	90.6±9.48	89.09-91.90
**Blood Pressure (mmHg)**		
Systolic	122±15.38	119.9-124.3
Diastolic	79.5±10.9	77.96-81.05
	**Median**	**IQR**
**Recent CD4 Count (cells/mm³)**	460	.[326.5, 633.5]

*TASH: Tikur Anbessa Speciaized Hospital, ZMH: Zewditu Memorial Hospital
†cART: combined Antiretroviral Therapy;**1a:** Stavudine (D4T)/ Lamuvidin (3TC) /Nevarapin(NVP); **1b:** D4T/3TC/ Efaverinez (EFV); **1c:** Zidovudine (ZDV)/3TC/ NVP; **1d:** ZDV/ 3TC/ EFV; **1e**: Tenofovir (TDF)/3TC/EFV; **1f:**TDF/3TC/NVP; **1g:**Abacavir(ABC)/3TC/EFV;**1h:** ABC/3TC/NVP; **2e:**AZT/3TC/ Ritonavir boosted Lopinavir (LPV/r); **2f:**AZT/3TC/ Ritonavir boosted Atazanavir (ATV/r)**; 2g:**TDF/3TC/ (LPV/r); **2h:**TDF/3TC/ ATV/r;**2i:** ABC/3TC/LPV/r
‡ Participants may have more than one reason for change of cART

PREVALENCE OF METABOLIC SYNDROME, LIPODYSTROPHY, HYPERTRIGLYCERIDEMIA, AND DISTRIBUTION OF LIPODYSTROPHY MANIFESTATIONS

In this cohort of 518 study participants, the prevalence of MetS by IDF 2006 criteria was found to be 193 (37.3%) and by ATPIII 2005 criteria 195(37.6%). The Prevalence of Lipodystrophy was 23.6% (122 cases). The prevalence of Hypertriglecridemia was 40.7% (211 cases).(Table 2) Central adiposity ±extremity wasting was the highest 80(15.4%) participants reported lipodystrophy manifestation followed by loss of buccal fat pad 76(14.7%). The highest lipodystrophy manifestation by physical exam was central adiposity ±extremity wasting 61(11.8%) followed by loss of buccal fat pad 60 (11.6%).(Table 2)

For peer review only

Table 2: Prevalence of metabolic syndrome, lipodystrophy, hypertriglyceridemia, and the distribution of lipodystrophy manifestations in HIV patients on cART* at TASH* and ZMH*, Addis Ababa, Ethiopia

Variables	Frequency	Percent
**Metabolic Syndrome IDF 2006**		
Yes	193	37.3
No	325	62.7
**Metabolic Syndrome ATP III 2005**		
Yes	195	37.6
No	323	62.4
**Lipodystrophy**		
**Participants reported**		
Yes	117	22.6
No	401	77.4
**Physical Exam**		
Yes	122	23.6
No	396	76.4
**Hypertrigleceridemia**		
Yes	211	40.7
No	307	59.3
**Lipodystrophy manifestations:**		
**Participants reported**		
**Loss of buccal fat Pad**		
Yes	76	14.7
No	442	85.3
**Central adiposity ±extremity wasting**		
Yes	80	15.4
No	438	84.6
**Supraclavicular/dorsocervical fat pad**		
Yes	39	7.5
No	479	92.5
**Breast Enlargement**		
Yes	31	6.0
No	486	93.8
**Lipodystrophy manifestations: Physical Examination**		
Loss of buccal fat pad	60	11.6
Central adiposity ±extremity wasting	61	11.8
Supraclavicular/dorsocervical fat pad	1	0.2
No Sign of Lipodystrophy	396	76.4

*cART: combined Antiretroviral Treatment, TASH: Tikur Anbessa Specialized Hospital, ZMH: Zewditu Memorial Hospital

ASSOCIATED RISK FACTORS FOR METABOLIC SYNDROME AND LIPODYSTROPHY

Bivariate regression analysis revealed a significant association with MetS between age ≥45 years [cHR=1.68, 95%CI = 1.1-2.5, p=0.009], female sex [cHR=1.71, 95%CI=1.2-2.5, p=0.003], BMI(kg/m^2) ≥ 25 [cHR=3.1, 95%CI = 2.1-4.4), p=0.001], and EFV based cART regimen [cHR = 2.8, 95%CI= 1.71-4.65, p=0.001] based on IDF 2006 criteria.

In contrast to the nevirapin (NVP) based-regimen, the lopinavir-based regimen had cHR = 2.8, 95% CI = 0.94-8.26. Independent risk factors for MetS with multivariable regression analysis were age ≥45 years [aHR=1.8, 95%CI= 1.2-2.4, p=0.006], female sex[aHR= 1.8, 95%CI= 1.1-2.8, p=0.013], BMI (kg/m^2) ≥ 25 [aHR= 2.71 , 95%CI= (1.8-4.1), p=0.001], EFV based cART regimen [aHR=2.8 95%CI = 1.6-4.8, p=0.001], and LPV based cART regimen [aHR= 3.7, 95%CI = 1.0-13.3), p=0.043]. CD4 count above 350 cells/mm^3 was not significantly associated with MetS in this study [cHR= 1.7, 95%CI = 0.8-3.6), p=0.143, aHR =1.5, 95%CI= 0.7-3.4%), p=0.320].(Table.3)

For peer review only - http://bmjopen.bmj.com/site/about/guidelines.xhtml

Table 3: Associated Risk Factors for Metabolic Syndrome among HIV patients on cART* at TASH* and ZMH*, Addis Ababa, Ethiopia

	Metabolic Syndrome †		cHR (95%CI)	P-Value	aHR (95%CI)	P-Value
**Variables**	Yes	No				
**Age (yrs)**						
**<45**	80(31.0%)	178(69.0%)	1		1	
**≥45**	113(43.5)	147(56.5%)	1.68 (1.14-2.49)	0.009	1.79(1.18 -2.37)	0.006
**Sex**						
**Male**	50 (29.4%)	120 (70.6%)	1		1	
**Female**	143(41.2%)	204 (58.8%)	1.71(1.19 -2.45)	0.003	1.79(1.13 - 2.83)	0.013
**Recent CD4 Count (cells/mm^3)**						
**<200**	11 (29.0%)	27 (71.0%)	1		1	
**200-350**	30 (29.4%)	72 (70.6%)	1.02 (0.45-2.32)	0.957	0.99(0.41-2.40)	0.989
**>350**	143(41.3%)	203(59.7%)	1.73(0.83-3.60)	0.143	1.50(0.67-3.35)	0.320
**BMI (kg/m^2)**						
**≤24.9**	83 (27.0%)	225(73.0%9	1		1	
**≥25**	108 (53.0%)	6 (47.0%)	3.05(2.10-4.43)	0.001	2.71(1.81-4.06)	0.001
**Current cART‡**						
**NVP based**	24(21.8%)	86(79.2%)	1		1	
**EFV based**	152(44.1%)	193(55.9%)	2.82(1.71-4.65)	0.001	2.79(1.62-4.80)	0.001
**LPV based**	7 (43.7%)	9 (56.3%)	2.79(0.94-8.26)	0.640	3.72(1.04-13.3)	0.043
**ATV based**	10 (21.3%)	37(78.7%)	0.97(0.42-2.23)	0.940	1.03(0.41-2.60)	0.954

* cART: combined Antiretroviral Treatment, TASH: Tikur Anbessa Specialized Hospital, ZMH: Zewditu Memorial Hospital

†Based on IDF 2006 Metabolic Syndrome Diagnostic Criteria

‡Current cART: NVP: Nevirapin, EFV:Efavirenz, LPV: Lopinavir, ATV: Atazanavir

Associated risk factors for Lipodystrophy on bivariate analysis were exposure to a stavudine containing regimen [cHR= 3.1, 95%CI= 1.7-5.7, p=0.001] and BMI(kg/m^2) ≥ 25 [cHR= 2.8, 95%CI= 1.8-4.2, p=0.001].. Age above 45 years was not found to be associated with lipodystrophy in our study(p=0.567). An independent risk factor for lipodytrophy was a cART regimen containing stavudin [aHR= 3.1, 95%CI= 1.6-6.1, p=0.001]. There was no significant

For peer review only

association between CD4 count and recent cART regimen and Lipodystrophy in this study.(Table 4)

Table 4: Associated Risk factors for Lipodystrophy among HIV Patients on cART* at TASH*and ZMH*, Addis Ababa, Ethiopia

	Lipodystrophy Sign(s)		cHR (95%CI)	*p*-value	aHR (95%CI)	*p*-Value
**Variables**	**Yes**	**No**				
**Initial cART†**						
**D4T Exposed**	36(52.2%)	33(47.8%)	3.09(1.68-5.68)	0.001	3.13(1.6-6.12)	0.001
**D4T Not exposed**	35(26.1%)	99(73.9%)	1		1	
**CD4 Count (cells/mm³)**						
**<200**	6(16.7%)	30(83.3%)	1		1	
**200-350**	18(17.3%)	86(82.7%)	1.14(0.42-3.14)	0.795	0.63(0.18-2.18)	0.462
**>350**	92(26.6%)	254(73.4%)	1.93(0.78-4.77)	0.153	0.62(0.27-1.41)	0.254
**BMI* (kg/m²)**						
**≤24.9**	71(34.7%)	133(65.2%)	1		1	
**≥25**	50(16.2%)	258(83.8%)	2.76(1.81-4.18)	0.001	1.48(0.76-2.86)	0.249
**Current cART‡**						
**NVP based**	24(21.8%)	86(78.2%)	1		1	
**EFV based**	85(24.6%)	260(75.4%)	1.17(0.70-1.96)	0.547	1.20(0.52-2.78)	0.666
**LPV based**	0	16(100%)	0.00(00-)	0.998	0.00(0.00-)	0.999
**ATV based**	13(27.7%)	34(72.3%)	1.37(0.63-3.00)	0.431	0.90(0.29-2.77)	0.857

*TASH: Tikur Anbessa Specialized Hospital, ZMH: Zewditu Memorial Hospital, BMI: Body Mass Index, cART: combined Antiretroviral Therapy
†Intial cART regimen: for those participants whose drugs were changed to the current cART regimen ; D4T : Stavudine
‡Current cART: NVP: Nevirapin, EFV:Efavirenz, LPV: Lopinavir, ATV: Atazanavir

DISCUSSION

Our study revealed the prevalence of MetS is 37.3% by IDF 2006 criteria and 37.6% by ATP III 2005 criteria.[18,19] In contrast to a MetS prevalence in an HIV-negative study population with hypertension in Northern Ethiopia by Tachebele et al.[24] [40.7% IDF and 39.3% ATPIII], our finding was slightly lower. The prevalence of MetS was higher with both criteria in our study than the 14% reported by Samaras and colleagues,[25] (males,72.8%) by IDF criteria and 18%

For peer review only - http://bmjopen.bmj.com/site/about/guidelines.xhtml

(males,84.9%) by ATPIII criteria. Berhane et al.[12] reported from Southern Ethiopia a prevalence of 21.1% by IDF criteria. The prevalence of MetS in our setting is higher than that reported in a Tanzanian study which revealed a 25.6% prevalence using IDF criteria.[26] This study's finding of the prevalence of MetS is lower than an Italian study that reported 45.4% prevalence by ATPIII criteria.[27] Compared with our study finding, the prevalence of MetS using IDF criteria was 4.8% (8.6% in females and 1.8% in males) in the national NCD STEPS survey in the Ethiopian general population.[28] Undoubtedly, our finding indicates a significantly higher risk of CVD among PWH receiving cART. The above discrepancies in the prevalence rates are partly explained by the time differences between the studies, the types of cART taken, and the populations studied. Even though the IDF criteria appears to be more stringent because of the requirement of waist circumference, it also allows more patients with lesser waist circumference to be included as was evident in this result indicating a closer prevalence to the ATP III criteria. Similarly, Samaras et al.[25] have found a higher prevalence of MetS by ATPIII criteria. A slightly higher value of MetS prevalence with IDF criteria was reported by Hirigo and Tesfaye in Southern Ethiopia, 24.3% and 17.8 % using the IDF and NCEP-ATP III criteria, respectively.[29]

Independent predictors of MetS in our study were age above 45 years, female sex, BMI (kg/m^2) above 25, EFV-based cART regimen, and LPV-based cART regimen. Similar to our findings, studies in Ethiopia and Tanzania have found a significant association between female sex and MetS.[29,26] This is in line with other reports.[25,30] In contrast, one study revealed that gender has no significant association with MetS.[31] Similar to our study findings, Hirigo and Tesfaye have reported an increasing age above 40 years to be significantly associated with MetS.[29] This is in line with the report from Spain that has shown an association between increasing age

For peer review only

and MetS.[25] Similar to our study finding, higher BMI was significantly associated with MetS in other studies.[25,29] Furthermore, several other studies illustrated that the BMI is a quantitative predictor of MetS.[32-34]

Current use of EFV- based regimens is significantly associated with MetS in our study. This is supported by the fact that nonucleoside reverse transcriptase inhibitors impair lipid metabolism as a class,[35] and efavirenz leads to increased inflammation,[36,37] impaired adipogenesis and adipocyte differentiation,[36] impaired metabolism,[36,37] and no mitochondrial toxicity.[37] Efavirenz use is associated with increased apoptosis, increased mitochondrial mass, and oxidative stress in hepatic cells. Disruption in mitochondrial membrane potential was observed in hepatocytes promoting cytochrome c release and apoptosis.[38,39] Efavirenz also inhibits complex I of the electron transport chain (ETC) stimulating ROS production and a decrease in ATP which leads to an increase in the lipid content of hepatic cells.[40] Efavirenz also caused increased mitochondrial depolarization and altered mitochondrial morphology leading to mitophagy (clearance of damaged mitochondria) in neuronal cells.[41] Mitochondrial dysfunction promotes the progression of MetS by encouraging the occurrence of inflammation and oxidative stress. Efavirenz also acts as a pregnane X receptor agonist that promotes dyslipidemia and hypercholesteremia.[42] Non-nucleoside reverse transcriptase inhibitors (NNRTIs) promote MetS by reducing insulin sensitivity through their pro-inflammatory effects. NNRTIs are known for their reduction of adiponectin concentrations through increased expression of TNF-a, IL-6, and IL-1B. The reduction in the insulin sensitivity modulator, adiponectin, allows for the progression of insulin resistance.[43] Current EFV treatment, has been correlated with elevated markers of atherosclerosis.[44] The finding of liponavir- based cART regimen is in line with the independent association with MetS in a study in Barcelona,

For peer review only

Spain.[25] This is supported by the molecular mechanism of the drug that causes substantial impairment of adipocyte differentiation and lipid or glucose metabolism, pronounced decrease in mitochondrial proteins, and increased inflammation.[45]

The prevalence of lipodystrophy in this study was found to be 22.6% by subjective participants report and 23.6% by objective assessment for lipodystrophy manifestations. Our study finding is higher than the report of 12.1% by Berhane et al.[12] from South West Ethiopia and lower than the 68.3% prevalence in the study from Addis Ababa.[13] In our study, the higher prevalence compared to Berhane et al.[12] finding might be attributable to the larger sample size and the lower prevalence compared to Feleke et al.[13] finding may be explained by the trends of cART drug prescription that has been towards withdrawal of Stavudine. In our study, the prevalence of hypertriglyceridemia ≥ 150 mg/dl, a marker of lipodystrophy with insulin resistance and a component of MetS, was found in 40.7% of participants. This finding is closer to the finding of 43.5% prevalence in urban Tanzanian HIV population on cART,[26] higher than the 15.2% prevalence finding in Addis Ababa,[13] and lower than the report of 59.6% prevalence in a study in defense hospital in Addis Ababa.[46] Unadjusted, Lipodystrophy was significantly associated with a Stavudine- containing regimen and BMI (kg/m^2) ≥ 25. However, after adjustment for CD4 count ($cells/mm^3$) and current cART, only D4T exposure was significantly associated with Lipodystrophy. This finding of strong association between D4T and lipodystrophy is in line with previous reports.[47] This is because of the nucleoside reverse transcriptase class effect on impairing lipid metabolism,[35] and D4T effect of reduction of mitochondrial DNA.[48,49] Caron etal.[50] in their in vitro study have shown that Stavudin leads to decreases in lipid content, survival of adipose cells, and mitochondrial activity. The amount of mtDNA was found to be significantly decreased among patients receiving NRTIs indicating the mechanistic effect

For peer review only

of Stavudin on mitochondrial toxicity.[51] The resultant inflammatory cytokines such as Interleukins results in adipose cell destruction. This is known to impair Insulin signaling function and glucose transport. ART-associated lipodystrophy increases the risk for dyslipidemia, insulin resistance, diabetes mellitus, and heart disease.[52]

Limitations of this study are the cross sectional design, difficulty to trace some of the records on the initial cART regimen from old records, difficulty to get adequate samples for some of the variables that may have influenced the outcome like patients on Atazanavir based regimen, and fewer patients who were smokers or taking alcohol to identify their potential correlation in the study participants, and fewer patients on Liponavir based regimen as well. Besides, a complete nutritional assessment was not made. Advanced techniques for the assessment of lipodystrophy were not utilized because of availability. Of all the studies done in Ethiopia on MetS and Lipodystrophy, this is the one with the largest sample size and laboratory test results available for all participants.

CONCLUSION

Metabolic Syndrome was diagnosed in 37.3% and 37.6% of PWH using IDF and ATPIII criteria respectively. The higher prevalence of MetS is associated with female sex, age above 45 years, BMI above 25 Kg/m^2, EFV- based and Liponavir-based cART regimens. Hypertriglyceridemia was common among PWH receiving cART indicating cardiovascular and metabolic risks. Lipodystrophy was detected in 23.6% of study participants. Previous stavudine exposure is significantly associated with lipodystrophy. These metabolic changes among those on cART place them at high risk of cardiovascular events. Therefore, screening for MetS in PWH receiving cART and managing modifiable CV risk factors are highly recommended.

For peer review only

Furthermore, a prospective cohort study is recommended to evaluate MetS and lipodystrophy and the related CV outcomes among patients on cART.

AUTHOR CONTRIBUTION

AA, IO, and WA conceptualized the study. AA collected the primary data, conducted the analysis, and drafted the manuscript. HY, AR, AS, IO, and WA contributed to conceptualization, collection of data, and data analysis. AA, HY, AR, AS, IO, and WA have read, reviewed, and approved the article.

FUNDING

This collaborative research work is financially supported by an NIH funded project, Medical Education Partnership Initiative- Junior Faculty (D43TW010143).

ACKNOWLEDGMENT

The authors would like to acknowledge the study participants for their voluntary participation, the data collectors and the study centers staff at Zewditu Memorial Hospital and Tikur Anbessa Specialized Referral Hospital, for their invaluable support in the conduct of our study. In addition, the authors acknowledge Ms. Dorqa Woldesenbet (MPH) from School of Public Health-Addis Ababa University for her support in the statistical analysis.

CONFLICT OF INTEREST

The authors declare that this research was conducted in the absence of any commercial or financial conflict of interest.

For peer review only

ETHICAL CONSIDERATIONS

The study has been reviewed and approved by the Institution Review Board of the College of Health Sciences- Addis Ababa University with the Ref/ID# 077/17/IM and by Addis Ababa city regional health bureau Ethics review committee and have therefore been performed in accordance with the ethical standards of Declaration of Helsinki. Information sheet detailing the procedure of the study, the benefits the of the study, the full right to agree or disagree to participate in the study, assurance of confidentiality, continuation of their usual medical care regardless of their agreement or disagreement to participate in the study, and the linkage to the treating team if there are clinical or lab findings that require attention was shared to study participants. For participants who cannot read or had questions, data collectors had read, responded, and explained. Written informed consent were obtained from all participants of the study.

Data Availability Statement:

The data for this study is available upon a reasonable request. Access to the data can be granted by contacting the corresponding author.

REFERENCES:

1. UNAIDS Global HIV & AIDS Statistics—2022 Fact Sheet. Available online: https://www.unaids.org/en/resources/fact-sheet. Accessed on 09/22/2023.
2. Kasper LD, Fauci SA, Hauser SL, Longo DL, Jameson LJ, Loscalzo; Harrison's Principles of Internal Medicine- 20th edition. 2018. New York. McGraw-Hill Medical Publishing Division.
3. IN DANGER: UNAIDS Global AIDS Update 2022. Geneva: Joint United Nations Programme on HIV/ AIDS; 2022. Licence: CC BY-NC-SA 3.0 IGO. Available on line:

For peer review only

https://www.unaids.org/sites/default/files/media_asset/2022-global-aids-update_en.pdf. Accessed on 09/22/23.

4. Obel N, Omland LH, Kronborg G *et al.* Impact of non-HIV and HIV risk factors on survival in HIV-infected patients on HAART: a population-based nationwide cohort study. *PLoS ONE*6(7), E22698 (2011). doi.org/10.1371/journal.pone.0022698

5. Data Collection on Adverse Events of Anti H. I. V. drugs Study Group, Smith C, Sabin CA, Lundgren JD, etal. Factors associated with specific causes of death amongst HIV-positive individuals in the D:A:D study. AIDS 2010; 24: 1537–1548. DOI:10.1097/QAD.0b013e32833a0918

6. Smit C, Geskus R, walker S, et al. effective therapy has altered the spectrum of cause specific mortality following HIV seroconversion. AIDS. 2006;20:741-749 doi: 10.1097/01.aids.0000216375.99560.a2. PMID: 16514305.

7. Samji H, Cescon A, Hogg RS, et al. North American AIDS Cohort Collaboration on Research and Design (NA-ACCORD) of IeDEA,Closing the Gap: increase in life expectancy among treated HIV positive individuals in the United States and Canada. PLoSone. 2013; 8(12):e81355 doi: 10.1371/journal.pone.0081355. PMID: 24367482; PMCID: PMC3867319.

8. Lars G. Hemkens ,Heiner C. Bucher; novel therapeutic concepts, HIV infection and cardiovascular disease; European Heart journal , REVIEW; advance access published January 9,2014; doi:10.1093/euroheartj/eht528

9. Grover SA, Coupal L, Gilmore N, Mukherjee J. Impact of dyslipidemia associated with highly active antiretroviral therapy oncardiovascular risk and life expectancy. Am J Cardiol. 2005 Mar 1;95(5):586-91. doi: 10.1016/j.amjcard.2004.11.004. PMID: 15721096.

For peer review only

10. Domingo P, Sambeat MA, Pérez A, Ordoñez J, Rodriguez J, Vázquez G. Fat distribution and metabolic abnormalities in HIV-infected patients on first combination antiretroviral therapy including stavudine or zidovudine: role of physical activity as a protective factor.AntivirTher. 2003; 8(3):223-231. doi: 10.1177/135965350300800306. PMID: 12924539.

11. Calza L, Manfredi R, Chiodo F. Dyslipidaemia associated with antiretroviral therapy in HIV-infected patients. J Antimicrob Chemother.2004;53(1):10-14. doi: 10.1093/jac/dkh013. Epub 2003 Nov 25. PMID: 14645323.

12. Berhane T , Yami A, Alemseged F,etal; prevalence of lipodystrophy and Metabolic syndrome among HIV positive individuals on HAART in Jimma, south west Ethiopia; Pan African Medical Journal. 2012; 13:43 Epub 2012 Oct 30. PMID: 23330034; PMCID: PMC3542806.

13. Feleke Y, Fekade D, Mezgebu Y; prevalence of HAART associated metabolic abnormalities and lipodystrophy in HIV infected patients; Ethiopian medical Journal:2012, 50(3):221-230 PMID: 23409405.

14. Federal Democratic republic of Ethiopia, Ministry of Health: health sector development program IV 2010/11-2014/15.Available at: https://www.healthynewbornnetwork.org/hnn-content/uploads/HSDP-IV-Final-Draft-October-2010-2.pdf. Accessed on 22/9/2023.

15. Gibson, R.S., Principles of nutritional assessment. *Introduction to Anthropometry. Available at: https://nutritionalassessment.org/intant/. Accessed on 09/22/2023.*

16. Waist Circumference and Waist–Hip Ratio: Report of a WHO Expert Consultation Geneva, 8–11 December 2008 Available at: https://iris.who.int/bitstream/handle/10665/44583/9789241501491_eng.pdf. Accessed on 09/22/2023..

For peer review only

17. Perloff D, Grim C, Flack J et al. Human blood pressure determination by sphygmomanometry. Circulation. 1993 Nov;88 (5 pt 1):2460-2470. doi: 10.1161/01.cir.88.5.2460. PMID: 8222141.

18. Alberti KG, Zimmet P, Shaw J. Metabolic syndrome--a new world-wide definition. A Consensus Statement from the International Diabetes Federation. Diabet Med. 2006 May;23(5):469-80. doi: 10.1111/j.1464-5491.2006.01858.x. PMID: 16681555.

19. Grundy SM, Brewer HB Jr, Cleeman JI, Smith SC Jr, Lenfant C; American Heart Association; National Heart, Lung, and Blood Institute. Definition of metabolic syndrome: Report of the National Heart, Lung, and Blood Institute/American Heart Association conference on scientific issues related to definition. Circulation. 2004; 109:433- 438. doi: 10.1161/01.CIR.0000111245.75752.C6. PMID: 14744958.

20. Freitas P, Santos AC, Carvalho Det al. Fat mass ratio: an objective tool to define lipodystrophy in HIV-infected patients under antiretroviral therapy. J Clin Densitom. 2010 Apr-Jun;13(2):197–203. doi: 10.1016/j.jocd.2010.01.005. Epub 2010 Mar 27. PMID: 20347364.

21. Bacchetti P, Gripshover B, Grunfeld C, et al. Study of Fat Redistribution and Metabolic Change in HIV Infection (FRAM). Fat distribution in men with HIV infection. J Acquir Immune Defic Syndr. 2005 Oct 1;40 (2):121–31.doi: 10.1097/01.qai.0000182230.47819.aa. PMID: 16186728; PMCID: PMC3166344.

22. Jantarapakde J, Phanuphak N, Chaturawit C, et al. Prevalence of metabolic syndrome among antiretroviral-naive and antiretroviral-experienced HIV-1 infected Thaiadults. AIDS Patient Care STDS. 2014Jul;28(7):331–40. doi: 10.1089/apc.2013.0294. Epub 2014 Jun 10. PMID: 24914459.

23. Carr A, Samaras K, Thorisdottir A, Kaufmann GR, Chisholm DJ, Cooper DA. Diagnosis, prediction, and natural course of HIV-1 protease-inhibitor associated lipodystrophy,

For peer review only

hyperlipidaemia, and diabetes mellitus: a cohort study. Lancet. 1999Jun;353(9170):2093–9. doi: 10.1016/S0140-6736(98)08468-2. PMID: 10382692.

24. Belaynesh Tachebele, Molla Abebe, Zelalem Addis, and Nebiyu Mesfin; Metabolic syndrome among hypertensive patients at University of Gondar Hospital, North West Ethiopia: a cross sectional study; BMC Cardiovascular Disorders 2014, 14:177. doi.org/10.1186/1471-2261-14-177

25. Samaras K, Wand H, Law M, Emery S, Cooper D, Carr A. Prevalence of metabolic syndrome in HIV-infected patients receiving highly active antiretroviral therapy using international diabetes foundation and adult treatment panel III criteria. Diabetes Care. 2007;30:113–9. doi.org/10.2337/dc06-1075

26. Kagaruki G, Kimaro G, Mweya C etal.; Prevalence and Risk Factors of Metabolic Syndrome among Individuals Living with HIV and Receiving Antiretroviral Treatment in Tanzania; British Journal of Medicine & Medical Research 5(10): 1317-1327, 2015, Article no.BJMMR.2015.150 ISSN: 2231-0614. DOI: 10.9734/BJMMR/2015/14455

27. Gazzaruso C, Sacchi P, Garzaniti A, Fratino P, Bruno R, Filice G: Prevalence of metabolic syndrome among HIV patients. Diabetes Care 2002Jul, 25(7):1253–1254. doi: 10.2337/diacare.25.7.1253. PMID: 12087038.

28. Gebreyes YF, Goshu DY, Geleta TK, Argefa TG, Zemedu TG, etal (2018); Prevalence of high blood pressure, hyperglycemia, dyslipidemia, metabolic syndrome and their determinants in Ethiopia: Evidences from the National NCDs STEPS Survey, 2015. PLOS ONE 13(5): e0194819. doi.org/10.1371/journal.pone.0194819

29. Agete Tadewos Hirigo, Demo Yemane Tesfaye; Influences of gender in metabolic syndrome and its components among people living with HIV virus using antiretroviral treatment in Hawassa, southern Ethiopia; *BMC Res Notes (2016) 9:145* DOI 10.1186/s13104-016-1953-2

For peer review only - http://bmjopen.bmj.com/site/about/guidelines.xhtml

30. Kolcić I, Vorko-Jović A, Salzer B, Smoljanović M, Kern J, Vuletić S Metabolic syndrome in a metapopulation of Croatian island isolates. Croat Med J. 2006Aug;47(4):585–92. PMID: 16909456; PMCID: PMC2080447.

31. Lauda LG, Mariath AB, Grillo LP. Metabolic syndrome and its components in HIV-infected individuals. Rev Assoc Med Bras(1992).. 2011;57(2):182-6. English, Portuguese. doi: 10.1590/s0104-42302011000200016. PMID: 21537705.

32. Jacobson DL, Tang AM, Spiegelman D, etal. Incidence of metabolic syndrome in a cohort of HIVinfected adults and prevalence relative to the US population (National Health and Nutrition Examination Survey). J Acquir Immune Defic Syndr. 2006 Dec;43 (4):458–66 doi: 10.1097/01.qai.0000243093.34652.41. PMID: 16980905.

33. Jericó C, Knobel H, Montero M, et al. Metabolic syndrome among HIV-infected patients: prevalence, characteristics, and related factors. Diabetes Care. 2005Jan;28(1):132-137. doi: 10.2337/diacare.28.1.132. PMID: 15616246.

34. Mondy K, Overton ET, Grubb J etal.. Metabolic syndrome in HIV-infected patients from an urban, midwestern US outpatient population. Clin Infect Dis. 2007 Mar 1;44 (5):726–34. doi: 10.1086/511679. Epub 2007 Jan 22. PMID: 17278068; PMCID: PMC3170426.

35. Minami R, Yamamoto M, Takahama S, Ando H, Miyamura T, Suematsu E. Comparison of the infl uence of four classes of HIV antiretrovirals on adipogenic diff erentiation: the minimal eff ect of raltegravir and atazanavir. *J Infect Chemother* 2011Apr; 17(2): 183–8.. doi: 10.1007/s10156-010-0101-5. Epub 2010 Aug 13. PMID: 20706762.

36. Diaz-Delfin J, Domingo P, Mateo MG, et al. Effects of rilpivirine on human adipocyte diff erentiation, gene expression, and release of adipokines and cytokines. *Antimicrob Agents Chemother* 2012 Jun;56 (6): 3369–75. doi: 10.1128/AAC.00104-12. Epub 2012 Mar 19. PMID: 22430974; PMCID: PMC3370806.

For peer review only - http://bmjopen.bmj.com/site/about/guidelines.xhtml

37. Diaz-Delfi n J, del Mar Gutierrez M, Gallego-Escuredo JM, et al. Eff ects of nevirapine and efavirenz on human adipocyte diff erentiation, gene expression, and release of adipokines andcytokines. *Antiviral Res* 2011Aug; 91(2): 112–19.. doi: 10.1016/j.antiviral.2011.04.018. Epub 2011 May 17. PMID: 21619898.

38. Jamaluddin M.S., Lin P.H., Yao Q., Chen C. Non-nucleoside reverse transcriptase inhibitor efavirenz increases monolayer permeability of human coronary artery endothelial cells. *Atherosclerosis.* 2010;208:104–111. doi: 10.1016/j.atherosclerosis.2009.07.029.

39. Ganta K.K., Mandal A., Chaubey B. Depolarization of mitochondrial membrane potential is the initial event in non-nucleoside reverse transcriptase inhibitor efavirenz induced cytotoxicity. *Cell Biol. Toxicol.* 2017;33:69–82. doi: 10.1007/s10565-016-9362-9.

40. Blas-García A., Apostolova N., Ballesteros D., Monleon D., Morales J.M., Rocha M., Victor V.M., Esplugues J.V. Inhibition of mitochondrial function by efavirenz increases lipid content in hepatic cells. *Hepatology.* 2010;52:115–125. doi: 10.1002/hep.23647.

41. Purnell P.R., Fox H.S. Efavirenz induces neuronal autophagy and mitochondrial alterations. *J. Pharmacol. Exp. Ther.* 2014;351:250–258. doi: 10.1124/jpet.114.217869.

42. Gwag T., Meng Z., Sui Y., Helsley R.N., Park S.-H., Wang S., Greenberg R.N., Zhou C. Non-nucleoside reverse transcriptase inhibitor efavirenz activates PXR to induce hypercholesterolemia and hepatic steatosis. *J. Hepatol.* 2019;70:930–940. doi: 10.1016/j.jhep.2018.12.038.

43. Lagathu C., Kim M., Maachi M., Vigouroux C., Cervera P., Capeau J., Caron M., Bastard J.-P. HIV antiretroviral treatment alters adipokine expression and insulin sensitivity of adipose tissue in vitro and in vivo. *Biochimie.* 2005;87:65–71. doi: 10.1016/j.biochi.2004.12.007.

44. Rudolph L. Gleason Jr., Alexander W. Cualk, Daniel Seifu, etal; Current Efavirenz (EFV) or Ritonavir-Boosted Lopinavir (LPV/r) Use Correlates with Elevate Markers of Atherosclerosis in

For peer review only - http://bmjopen.bmj.com/site/about/guidelines.xhtml

HIV-Infected Subjects in Addis Ababa, Ethiopia; PLOS ONE | DOI:10.1371/journal.pone.0117125 April 27, 2015

45. Capel E, Auclair M, Caron-Debarle M, Capeau J. Effects of ritonavirboosteddarunavir, atazanavir and lopinavir on adipose functionsand insulin sensitivity in murine and human adipocytes.*Antivir Ther* 2012; 17 (3): 549–56. doi: 10.3851/IMP1988. Epub 2011 Nov 28. PMID: 22293506.

46. Bayenes H, Ahmed M, Shenkute T, etal.;Prevalence and predictors of dyslipidemia on HAART and HAART naive HIV positive persons in Defense Hospital, Addis Ababa, Ethiopia; American Journal of Health Research.2014; 2(5): 303-309, doi: 10.11648/j.ajhr.20140205.23

47. Finkelstein JL, Gala P, Rochford R Glesby MJ and MehtaS.HIV/AIDS and lipodystrophy: Implications for clinical management in resource-limited settings; Journalof the International AIDS Society 2015 Jan 15; 18(1):19033|. doi: 10.7448/IAS.18.1.19033. PMID: 25598476; PMCID: PMC4297925

48. Feeney ER, van Vonderen MG, Wit F, et al. Zidovudine/lamivudine but not nevirapine in combination with lopinavir/ritonavir decreases subcutaneous adipose tissue mitochondrial DNA. *AIDS* 2012 Nov 13; 26 (17): 2165–74. doi: 10.1097/QAD.0b013e328358b279. PMID: 22874517.

49. McComsey GA, Daar ES, O'Riordan M, et al. Changes in fat mitochondrial DNA and function in subjects randomized to abacavir-lamivudine or tenofovir DF-emtricitabine with atazanavirritonavir or efavirenz: AIDS Clinical Trials Group study A5224s, substudy of A5202. *J Infect Dis* 2013 Feb 15; 207 (4): 604–11. doi: 10.1093/infdis/jis720. Epub 2012 Nov 29. PMID: 23204164; PMCID: PMC3549598.

50. Caron M, Auclair M, Lagathu C, et al. The HIV-1 nucleoside reverse transcriptase inhibitors stavudine and zidovudine alter adipocyte functions in vitro. AIDS. 2004;18(16):2127–2136. doi:10.1097/ 00002030-200411050-00004

For peer review only

51. Nolan D, Hammond E, Martin A, et al. Mitochondrial DNA depletion and morphologic changes in adipocytes associated with nucleoside reverse transcriptase inhibitor therapy. AIDS. 2003;17(9):1329–1338. doi:10.1097/00002030-200306130-00007

52. Fiorenza CG, Chou SH, Mantzoros CS. Lipodystrophy: pathophysiology and advances in treatment. Nat Rev Endocrinol. 2011;7 (3):137–150. doi:10.1038/nrendo.2010.199

For peer review only

Reporting checklist for cross sectional study.

Based on the STROBE cross sectional guidelines.

Instructions to authors

Complete this checklist by entering the page numbers from your manuscript where readers will find each of the items listed below.

Your article may not currently address all the items on the checklist. Please modify your text to include the missing information. If you are certain that an item does not apply, please write "n/a" and provide a short explanation.

Upload your completed checklist as an extra file when you submit to a journal.

In your methods section, say that you used the STROBE cross sectionalreporting guidelines, and cite them as:

von Elm E, Altman DG, Egger M, Pocock SJ, Gotzsche PC, Vandenbroucke JP. The Strengthening the Reporting of Observational Studies in Epidemiology (STROBE) Statement: guidelines for reporting observational studies.

		Reporting Item	Page Number
Title and abstract			
Title	#1a	Indicate the study’s design with a commonly used term in the title or the abstract	2
Abstract	#1b	Provide in the abstract an informative and balanced summary of what was done and what was found	2
Introduction			
Background / rationale	#2	Explain the scientific background and rationale for the investigation being reported	4
Objectives	#3	State specific objectives, including any prespecified hypotheses	5
Methods			
Study design	#4	Present key elements of study design early in the paper	6
Setting	#5	Describe the setting, locations, and relevant dates, including periods of recruitment, exposure, follow-up, and data collection	6
Eligibility criteria	#6a	Give the eligibility criteria, and the sources and methods of selection of participants.	6

For peer review only

	#7	Clearly define all outcomes, exposures, predictors, potential confounders, and effect modifiers. Give diagnostic criteria, if applicable	8
Data sources / measurement	#8	For each variable of interest give sources of data and details of methods of assessment (measurement). Describe comparability of assessment methods if there is more than one group. Give information separately for for exposed and unexposed groups if applicable.	6,7,8
Bias	#9	Describe any efforts to address potential sources of bias	7
Study size	#10	Explain how the study size was arrived at	7
Quantitative variables	#11	Explain how quantitative variables were handled in the analyses. If applicable, describe which groupings were chosen, and why	9
Statistical methods	#12a	Describe all statistical methods, including those used to control for confounding	9
Statistical methods	#12b	Describe any methods used to examine subgroups and interactions	9
Statistical methods	#12c	Explain how missing data were addressed	n/a
Statistical methods	#12d	If applicable, describe analytical methods taking account of sampling strategy	n/a
Statistical methods	#12e	Describe any sensitivity analyses	n/a
Results			
Participants	#13a	Report numbers of individuals at each stage of study—eg numbers potentially eligible, examined for eligibility, confirmed eligible, included in the study, completing follow-up, and analysed. Give information separately for for exposed and unexposed groups if applicable.	10
Participants	#13b	Give reasons for non-participation at each stage	n/a
Participants	#13c	Consider use of a flow diagram	n/a
Descriptive data	#14a	Give characteristics of study participants (eg demographic, clinical, social) and information on exposures and potential confounders. Give information separately for exposed and unexposed groups if applicable.	10, 11
Descriptive data	#14b	Indicate number of participants with missing data for each variable of interest	11
Outcome data	#15	Report numbers of outcome events or summary measures. Give information separately for exposed and	12,13,14

For peer review only

		unexposed groups if applicable.	
Main results	#16a	Give unadjusted estimates and, if applicable, confounder-adjusted estimates and their precision (eg, 95% confidence interval). Make clear which confounders were adjusted for and why they were included	14,15,16
Main results	#16b	Report category boundaries when continuous variables were categorized	n/a
Main results	#16c	If relevant, consider translating estimates of relative risk into absolute risk for a meaningful time period	n/a
Other analyses	#17	Report other analyses done—e.g., analyses of subgroups and interactions, and sensitivity analyses	n/a
**Discussion**			
Key results	#18	Summarise key results with reference to study objectives	17,18,19
Limitations	#19	Discuss limitations of the study, taking into account sources of potential bias or imprecision. Discuss both direction and magnitude of any potential bias.	19
Interpretation	#20	Give a cautious overall interpretation considering objectives, limitations, multiplicity of analyses, results from similar studies, and other relevant evidence.	20
Generalisability	#21	Discuss the generalisability (external validity) of the study results	20
**Other Information**			
Funding	#22	Give the source of funding and the role of the funders for the present study and, if applicable, for the original study on which the present article is based	20

None The STROBE checklist is distributed under the terms of the Creative Commons Attribution License CC-BY. This checklist can be completed online using https://www.goodreports.org/, a tool made by the EQUATOR Network in collaboration with Penelope.ai

For peer review only
